# Impact of Halide (Cl vs I) Identity on the Preferred Positioning of Substituents between Al and M (M = Co, Rh, Ir) in PAlP Pincer Complexes

**DOI:** 10.1021/acs.organomet.4c00490

**Published:** 2024-12-31

**Authors:** Samuel R. Lee, Natchayatorn Keawkla, R. Noah Sladek, Nattamai Bhuvanesh, Panida Surawatanawong, Oleg V. Ozerov

**Affiliations:** † Department of Chemistry, Texas A&M University, 3255 TAMU, College Station, Texas 77842, United States; ‡ Department of Chemistry and Center of Excellence for Innovation in Chemistry, Faculty of Science, Mahidol University, Bangkok 10400, Thailand

## Abstract

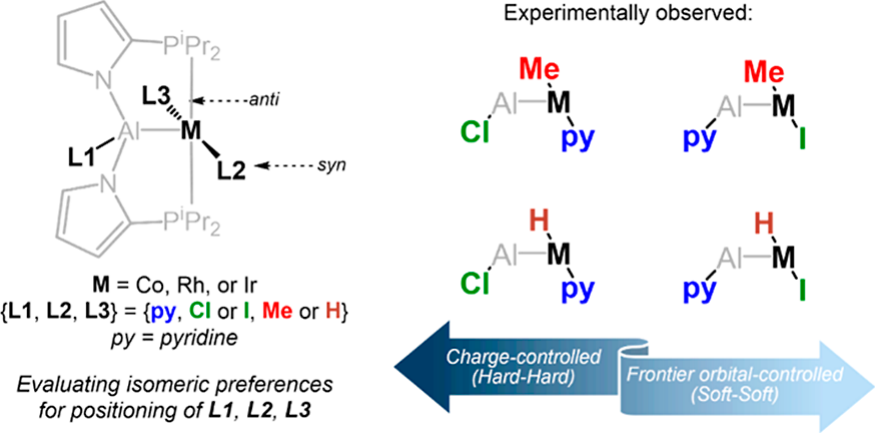

Protolysis of AlMe_3_ or AlEt_3_ with 2-diisopropylphosphinopyrrole (**1**) resulted in alane/bis(phosphine) pincer ligands containing two flanking phosphines and a central Al–Me (**2-Me**), Al–Et (**2-Et**) unit. Reactions of **2-Me** with [(COD)MI]_2_ (COD = 1,5-cyclooctadiene; M = Rh or Ir) in the presence of pyridine produced pincer complexes (**3–Rh–I** and **3–Ir–I**) with M supported by the PAlP tridentate ligand, and pyridine, methyl, and iodide as monodentate ligands for Al or M. The analogous reaction of **2-Et** with [(COD)MI]_2_ and pyridine resulted in the formation of the analogous compounds **4–Rh–I** and **4–Ir–I** with hydride in place of methyl. DFT calculations were used to analyze the thermodynamic preferences for the positioning of pyridine, methyl or hydride, and the halide (chloride or iodide) on M vs Al. Cobalt was included with Rh and Ir among M for the purposes of DFT calculations. Theoretical studies suggested that different isomers are preferred for the iodide complexes (M–I and Al–Py) than for the chloride ones (M–py and Al–Cl, previously reported for Rh and Ir). X-ray structural study of **3–Rh–I** and analysis of the ^1^H NMR data of the Rh and Ir compounds in benzene corroborated these predictions.

## Introduction

**Figure 1:**
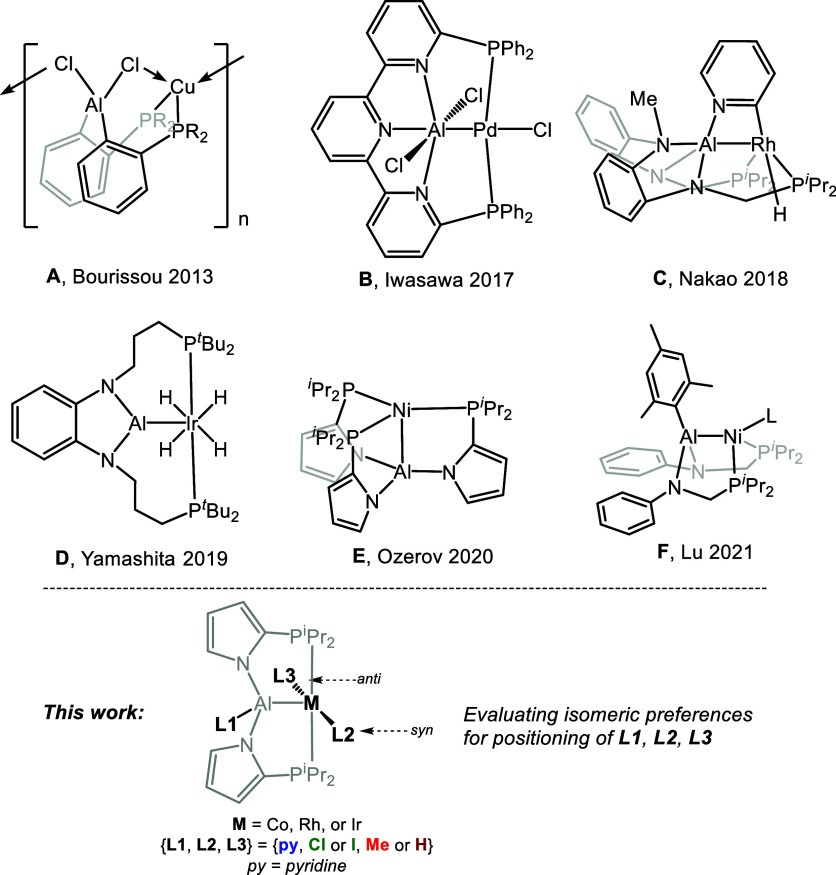
Selected previously reported complexes **A–F** of multidentate ligands with a central aluminum or boron anchor, and the generic labeling of the compounds in this work.

Polydentate ligands containing a central Al-based moiety have been attracting increasing interest as supporting frameworks for transition metals. The nature of the aluminum moiety and the mode of its interaction with the transition metal center can vary significantly, and this variation highlights the many possibilities for structural and reactivity discoveries. For example (Figure 1), compound **A** is a copper complex with an aluminate,^1^ compounds **B**, **C**, and **D** represent complexes of aluminyls (X-type)^2^ with different coordination numbers at Al,^3−5^ while **E** and **F** contain an alane (Z-type)^2,6^ moiety interacting with a transition metal.^7,8^ The electropositive nature of Al (relative to most transition metals) makes for an unusual M^δ−^–Al^δ+^ polarization, especially in aluminyl complexes.^9−13^ Already, transition metal complexes of Al-centered polydentate ligands have demonstrated potential as catalysts. Compounds with an alane moiety have been used by the Peters group in N_2_ reduction (with Fe),^14^ and by the Lu group (with Rh) in the studies of aryl-fluoride cleavage.^15^ Rh complexes with an aluminyl similar to **C** have been used by the Nakao group in C–H olefination of pyridines,^4,16^ magnesiation of aryl fluorides,^17^ and borylation of aryl ethers via C–O cleavage.^18,19^

Our group has been exploring the chemistry of aluminyl and alane complexes as an extension of our interest in the reactivity of boryl/bis(phosphine) PBP pincer ligands.^20−27^ In particular, we recently reported PAlP complexes of Rh and Ir supported by a bipodal ligand (bottom of Figure 1) that shares a pyrrole linker with **E** as a structural element.^28,29^ An important feature of the reactivity of these complexes is the apparent facility with which methyl, hydride, or chloride ligands could migrate between Al and Rh/Ir, depending on the thermodynamic preference. However, in those chloride complexes,^29^ we only observed pyridine bound to Rh/Ir, not Al. We became curious whether this preference can be reversed by using a softer base; iodide in place of chloride, and enable the formation of different isomers. We report the combination of our computational and experimental findings here.^30^

## Results and Discussion

### DFT Analysis

**Figure 2:**
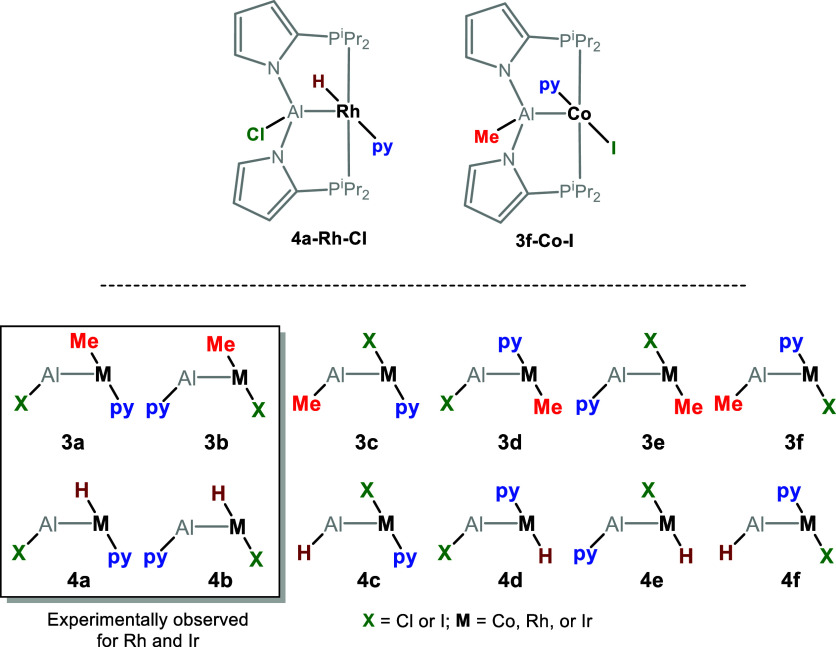
(Bottom) abbreviated structures of all isomers of **3** and **4** considered in this study. (Top) two representative examples of isomers shown in full. NB: the notation “**4a–Rh–Cl**” is meant to indicate the presence of both Rh and Cl in the molecule, but not necessarily the presence of a Rh–Cl bond.

We set out to analyze the isomeric preferences of the complexes **3–M–X** and **4–M–X** (Figure 2). For each formulation, keeping the (PAlP)M connectivity intact, there are three ligands/substituents that can be connected to either Al or the transition metal: pyridine, the halide (X), and methyl (**3** series) or hydride (**4** series). This gives rise to three possible connectivity patterns, each of which corresponds to a pair of diastereomers (syn vs anti), for a total of six isomeric structures (Figure 2). We then performed ground state optimization calculations for these hexads using M06/SDD/6-311G(d,p) and solvent-correction free energies in toluene using M06/SDD/6-311+G(d,p) (see Supporting Information for details). Each of the six combinations of the three metals of group 9 (Co, Rh, Ir) and the two halides under study (Cl or I) were explored. Although we have not prepared any Co complexes in this system yet, we decided to include Co in the computational study for the sake of completeness.

The calculated free energies of the isomers are presented in Tables 1 and 2, relative to the most stable isomer in a hexad (**a–f**) at zero. For the chloride complexes with all three metals, the lowest energy isomers are the **3a** and **4a** isomers, consistent with the experimental observations for Rh and Ir. On the other hand, when the halide is iodide, the lowest energy isomers are the **3b**/**4b** isomers, except for **3f–Co–I** that is lower in energy than **3b–Co–I** by 2.1 kcal/mol. Considering only the **a**/**b** isomer pairs, the system prefers chloride on Al and pyridine on M (**a**), but flips to preferring iodide on M and pyridine on Al (**b**). This flip corresponds to a change of 5–9 kcal/mol in relative preference. Among all isomers **a–f**, isomers **b** are second lowest in energy for the chloride compounds with a hydride (**4-Cl**). For the chloride compounds with a Me (**3-Cl**), the second lowest energy isomers are **3f–Cl** for Co and **3d–Cl** for Rh and Ir; however, the **3b–Cl** isomers are within <2 kcal/mol in energy of these.

**Table 1:** Solvent-Corrected Relative Free Energies (*ΔG*_rel_) of Isomers in the **3** Series (in kcal mol^–1^)

**M**	**3a**	**3b**	**3c**	**3d**	**3e**	**3f**
**X** = **Cl**						
Co	0.0	4.3	7.5	5.4	11.9	3.4
Rh	0.0	6.9	14.3	5.6	14.8	9.8
Ir	0.0	7.3	17.8	5.9	15.3	13.3
**X** = **I**						
Co	2.6	2.1	8.3	8.9	12.0	0.0
Rh	2.2	0.0	9.0	8.5	12.0	2.5
Ir	1.5	0.0	12.3	8.5	10.3	5.6

**Table 2:** Solvent-Corrected Relative Free Energies (*ΔG*_rel_) of Isomers in the **4** Series (in kcal mol^–1^)

**M**	**4a**	**4b**	**4c**	**4d**	**4e**	**4f**
**X** = **Cl**						
Co	0.0	4.8	27.7	7.7	22.1	24.9
Rh	0.0	6.5	35.2	9.3	27.0	32.7
Ir	0.0	7.7	38.5	10.7	24.8	36.6
**X** = **I**						
Co	3.4	0.0	30.3	14.2	26.9	23.3
Rh	4.4	0.0	34.7	16.7	20.3	28.2
Ir	7.8	0.0	39.1	18.8	19.4	33.1

Qualitatively, the preference for isomer **a** with chloride, and for isomer **b** with iodide, can be rationalized via the hard–soft acid–base framework: the harder acid (Al) has a higher preference for the harder base (Cl), while a softer acid (M) has a higher preference for the softer base (I). We set out to delve into this using the calculated natural Coulomb electrostatics (NCE) analysis (Table S1) and the second-order perturbation energy from NBO analysis (Table S2). The hard–hard interaction is charge controlled, while the soft–soft interaction is frontier orbital controlled. Consistent with these notions, the electrostatic interaction between Al and Cl in **3a–Rh–Cl** is significantly stronger than that between Al and I in **3a–Rh–I** and that between Al and pyridine in **3b–Rh–Cl** (Table S1), while the donor to acceptor orbital interaction from I to Rh in **3b–Rh–I** is stronger than that from Cl to Rh in **3b–Rh–Cl** and that from N of pyridine to Rh in **3a–Rh–I** (Table S2). It can also be noted that Al possesses a higher positive charge in the **a** isomers with the Al–Cl bond than in the **a** isomers with the Al–I bond (Table S6), and that the electrostatic potential at Al is significantly more positive than at Rh (Table S8 and Figure S3). However, these analyses did not allow for a quantitative basis for the calculated (and observed) preferences among the **a**/**b** isomers.

Instead, we resorted to a simpler analysis. Isomers **a** and **b** differ by a switch of the positions of the pyridine and the halide ligands. We examined the sum of the calculated Al–X and M–N distances in the **a** isomers (∑_**a,X**_) vs the sum of the Al–N and M–X distances in the **b** isomers (∑_**b,X**_, Table S5 and Figure S1). We reasoned that since both ∑_**a,X**_ and ∑_**b,X**_ can be viewed as sums of the same four covalent radii, a smaller sum should indicate a more advantageous bonding situation. For chloride complexes **3**/**4** of Ir and Rh, ∑_**a,Cl**_ < ∑_**b,Cl**_, suggesting that **3a–Cl/4a–Cl** is more effective for both group 9 metals. However, for the iodide complexes, ∑_**a,I**_ > ∑_**b,I**_ in **3**/**4** of Ir and Rh. This suggests that the combination of two bonds in **3b–I**/**4b–I** compounds is more effective. The differences between these sums correlate well to the calculated differences in free energy (Δ*G*(**b**–**a**), Figure S1).

An analogous analysis was also insightful for the comparison of isomers **a** and **f**. In this pairing, all three ligands (the halide, Me/H, and pyridine) change positions, although pyridine remains bound to M but switches between the **L2** and **L3** positions. We found that the energetic difference between an **a**/**f** isomer pair correlates very well with the calculated difference between the sums of the three requisite bond lengths in isomer **a** vs isomer **f** (Figure S5).

Other general observations can be made. The highest energy structures are those with a hydride on Al (**4c** and **4f**). The structures with a hydride in the **L2** (syn, Figure 1) position (**4d** and **4e**) are also relatively high in energy. Clearly, there is a strong preference for a hydride bound to the transition metal, and specifically in the **L3** (anti) position. The analogous preference for keeping the Me group in the **L3** position mostly holds (**3c**, **3d**, and **3e** are higher in energy), but it is smaller in magnitude than for the hydride. Moreover, the **3f** structures (Me on Al) are much closer in energy to **3a**/**3b** (Me on M) than **4f** (H on Al) structures are to **4a**/**4b** (H on M), evincing that there is less preference for placing Me on Al vs M than for placing H on Al vs M (H on Al is too unfavorable). This is consistent with the generally substantially greater strength of M–H vs M–Me bond for transition metals.^31^ It is especially true for Co, to the point that **3f–Co–I** is the lowest energy structure in its hexad. It can also be said that the magnitude of the differences among the isomers are mostly greatest for Ir, which is probably a reflection of Ir possessing strongest metal–ligand bonds. We considered whether Me/H in the L3 position on M can also interact weakly with Al and thus contribute to the stability of **3a**/**4a** and **3b**/**4b**. Indeed, the Wiberg bond indices for these Al–Me/H interactions in **3a**/**4a** and **3b**/**4b** were calculated to range from 0.09 to 0.28 (Tables S11 and S12). They are higher for Al–H over Al–Me, higher in **b** isomers over **a** isomers, and increase in the order of Ir < Rh < Co. In addition, we cannot exclude that the presence of pyridine and the halide in the syn **L1**/**L2** positions may allow an additional stabilizing interaction between the aryl C–H and the halide^32^ in **a** and **b** isomers. For example, the corresponding CH···Cl distances were calculated to be ca. 2.45–2.55 Å in the **3b–M–Cl** series (Table S4).

### Synthesis of Iodo-Containing PAlP Ir and Rh Complexes

**Scheme 1:**
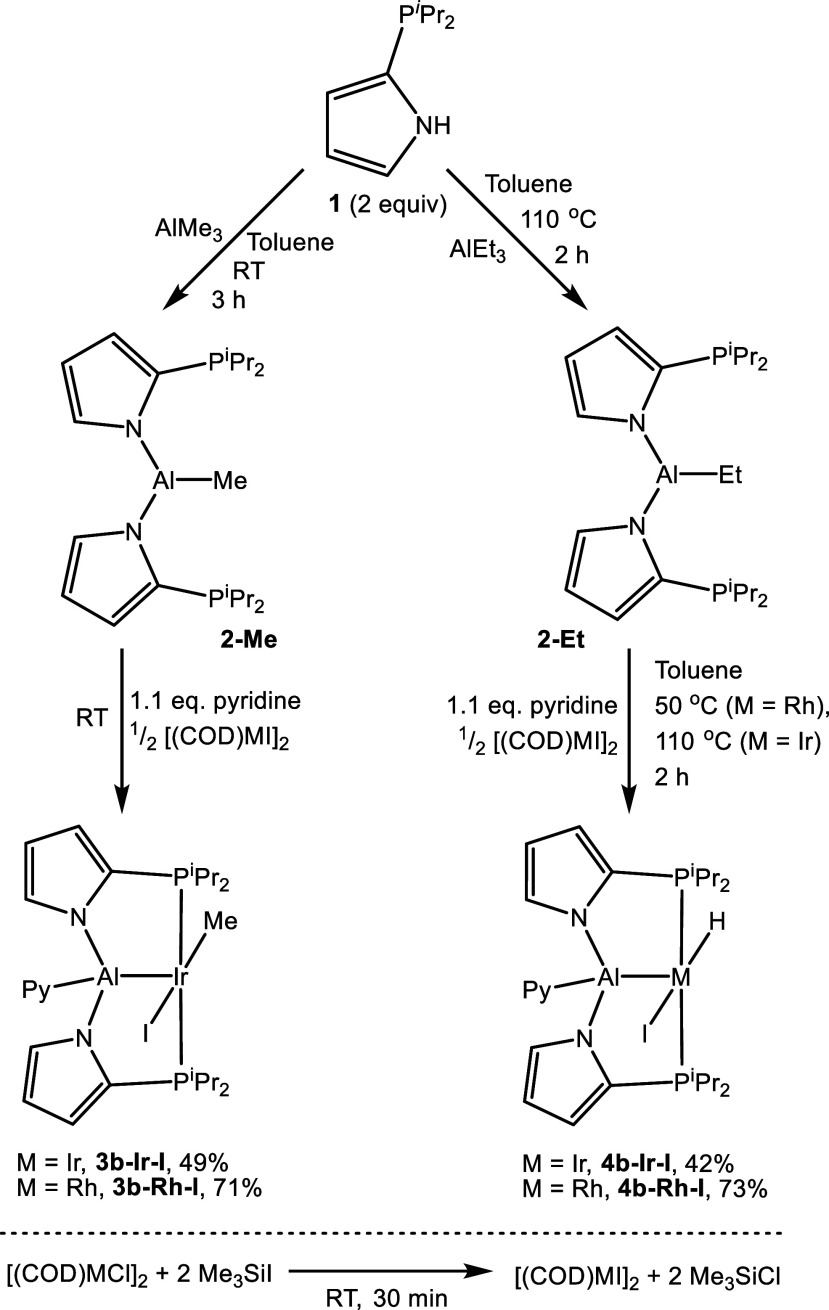
Synthesis or Rh and Ir Complexes **3** and **4**

We previously disclosed the syntheses of compounds **3a–M–Cl** and **4a–M–Cl** via reactions of PAl(R)P ligands with [(COD)MCl]_2_ precursors (M = Rh or Ir).^29^ In this work, we analogously utilized the [(COD)MI]_2_ precursors, generated in situ by reaction of [(COD)MCl]_2_ with Me_3_SiI (Scheme 1). To prepare the Me-containing complexes **3–Rh–I** and **3–Ir–I**, we used [(COD)MI]_2_ in combination with the PAl(Me)P ligand **2-Me**, which we previously reported.^29^ These reactions proceeded at ambient temperature. For the generation of the hydride complexes **4–Rh–I** and **4–Ir–I**, we utilized **2-Et** instead of the previously reported^29^**2-**^**i**^**Bu**.; this required thermolysis at 50 and 110 °C, respectively. Presumably, **2-Et** serves as a hydride precursor via formation of a M–Et intermediate and subsequent β-H elimination.

### Isomer Assignment

**Figure 3:**
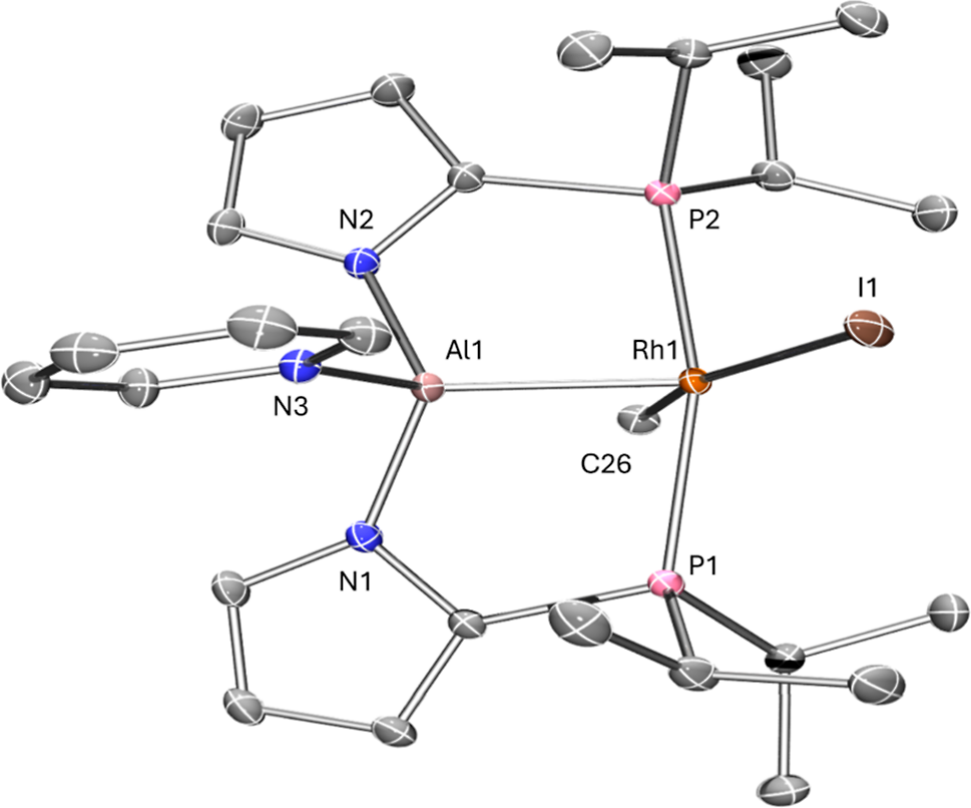
POV-ray rendition of the ORTEP drawing (50% thermal ellipsoids) of **3b–Rh–I** showing selected atom labeling, hydrogen atoms excluded. Selected bond distances (Å) and angles (deg) for **3b–Rh–I**: Rh1–Al1, 2.2934(3); Rh1–P1, 2.3412(3); Rh1–P2, 2.3382(3); Rh1–I1, 2.71498(10); Rh1–C1, 2.0982(10); Al1–N1, 1.8683(9); Al1–N2, 1.8665(9); Al1–N3, 1.9852(10); P1–Rh1–P2, 163.230(9); Al1–Rh1–P1, 82.316(10); Al1–Rh1–P2, 84.487(10); Al1–Rh1–I1, 121.922(9); Al1–Rh1–C1, 75.01(3); I1–Rh1–C1, 163.07(3); Rh1–Al1–N1, 109.81(3); Rh1–Al1–N2, 108.92(3); Rh1–Al1–N3, 117.58(3); N1–Al1–N3, 98.23(4); N2–Al1–N3, 101.25(4).

We were able to obtain a single crystal of **3–Rh–I** suitable for an X-ray diffraction study, which revealed the isomer **3b–Rh–I** (Figure 3), as predicted by DFT calculations. In spite of the halide/pyridine switch, the angles associated with the coordination environments about Al and Rh are similar for **3b–Rh–I** and for the previously reported **3a–Ir–Cl**.^33^ The P–M–P angles differ by less than a degree. The Me–M–Al angle is slightly more acute in **3b–Rh–I** (ca. 75°) than in **3a–Rh–Cl** (ca. 80°) or **3a–Ir–Cl** (ca. 82°). Interestingly, **3b–Rh–I** possesses the shortest known Rh–Al bond at ca. 2.29 Å,^34^ which is 0.03–0.06 Å shorter than the M–Al bonds in the structures of **3a–Rh–Cl**, **3a–Ir–Cl** and **4a–Rh–Cl**. These features are very well reproduced in the DFT-calculated structure of **3b–Rh–I** (Me–Rh–Al angle of 72.2°, P–Rh–P angle of 163.8°, Rh–Al bond distance of 2.312 Å).

**Figure 4:**
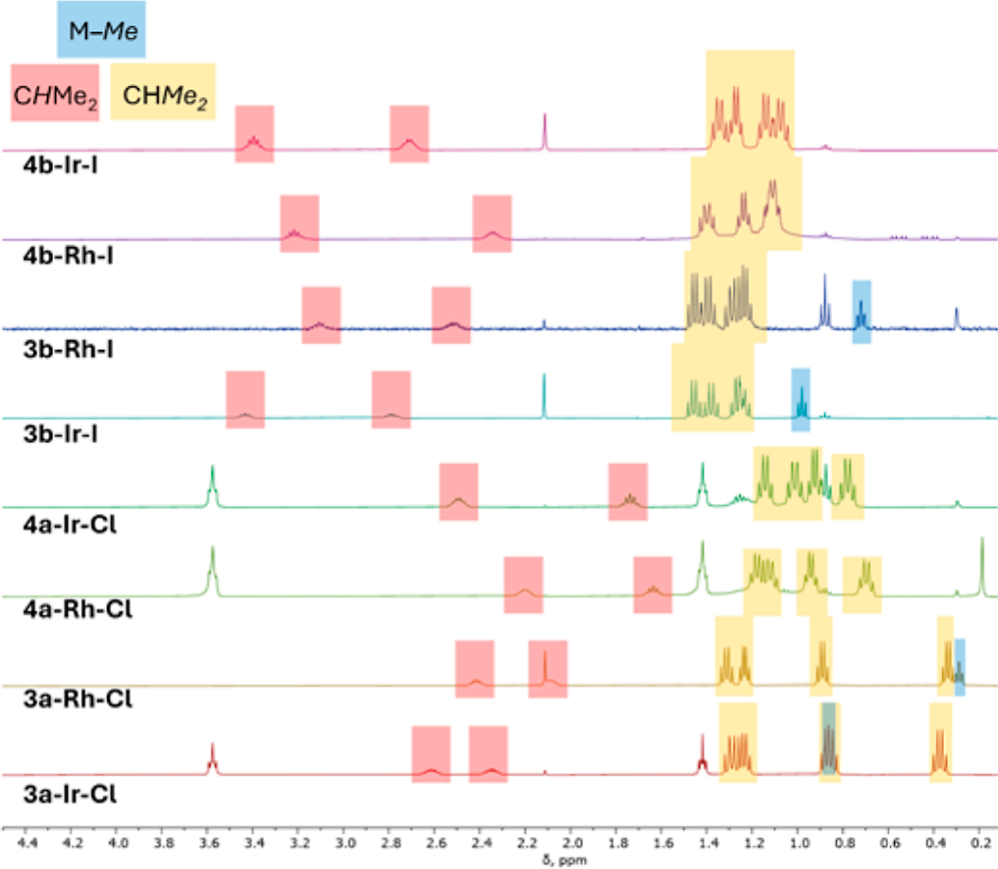
Comparison of aliphatic ^1^H NMR resonances for compounds **3** and **4**.

**Figure 5:**
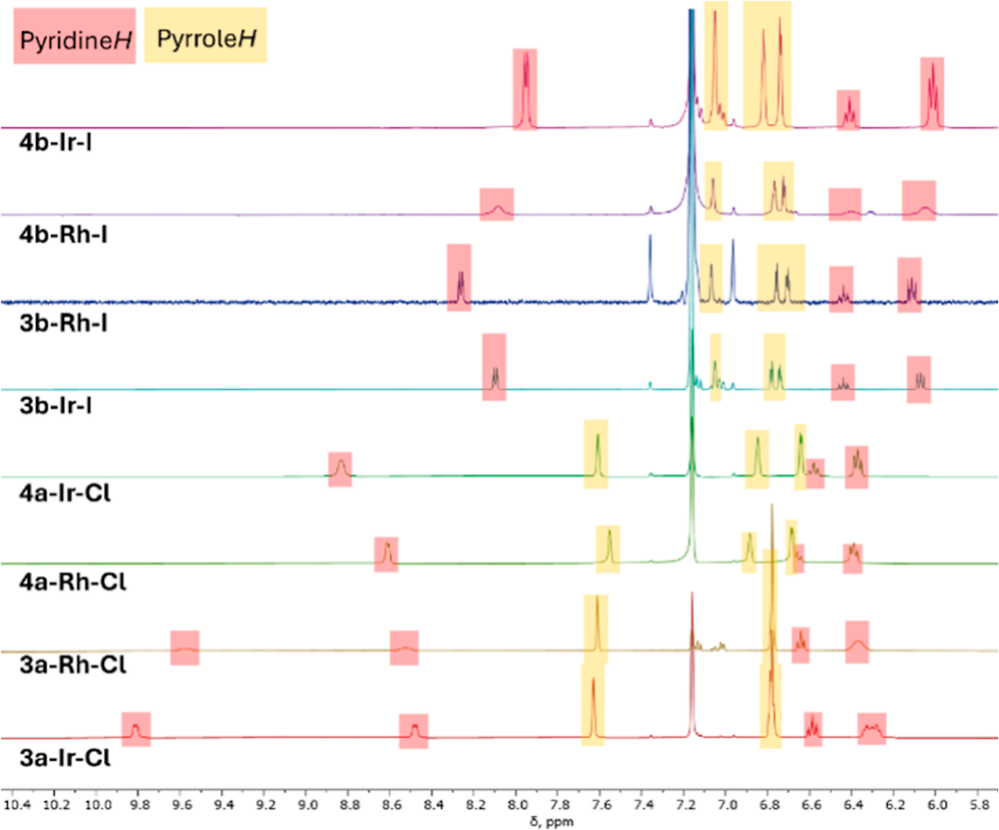
Comparison of aromatic ^1^H NMR resonances for compounds **3** and **4**.

^1^H NMR spectral data (Figures 4 and 5) were used to understand the isomeric nature of the iodide compounds in this study. We previously discussed that the placement of pyridine in the L2 position (isomers **3a/4a**) causes an upfield shift of some of the methyl resonances of the −P^i^Pr_2_ groups, because of the ring current effect of the pyridine ring. In addition, this “sandwiching” of the pyridine ring in L2 between two −P^i^Pr_2_ groups slows down the rotation about the M–N(pyridine) bond, leading to the loss of 2:2:1 symmetry among the pyridine resonances, or at least their broadening. Both of these manifestations were more pronounced for the M–Me complexes **3a–Rh–Cl** and **3a–Ir–Cl** than for the hydride complexes **4a–Rh–Cl** and **4a–Ir–Cl**.

As can be seen in Figure 4, all the complexes display four inequivalent methyl resonances and two inequivalent methine resonances (from the P^i^Pr_2_ groups). For all the chloride complexes, two of the methyl resonances are notably shifted upfield from their “normal” positions of >1.0 ppm. This is more pronounced for the M–Me complexes **3a–M–Cl**, but is also noticeable for **4a–M–Cl**. In contrast, none of the methyl resonances of P^i^Pr_2_ are upfield of 1.0 ppm for any of the iodide complexes, and there is less difference between the M–Me complexes and the M–H complexes in this regard. As well, the methine resonances for the iodide complexes are clustered at different chemical shifts than the methine resonances of the chloride complexes.

Considering the pyridine resonances in Figure 5, there are also clear differences in the patterns between the chloride complexes **3**/**4a–M–Cl** and the iodide complexes **3**/**4b–M–I**. The M–Me complexes **3a–Rh–Cl** and **3a–Ir–Cl** display inequivalent 2-CH resonances downfield of 8.4 ppm (averaging to around 9 ppm). **4a–Rh–Cl** and **4a–Ir–Cl** show only one such resonance of intensity 2H, but it is also downfield shifted to 8.6–8.8 ppm and is broadened. The positions of the other hydrogens of pyridine in the chloride complexes are very similar to each other in the 6.3–6.7 ppm range. On the other hand, the 2-CH resonances of pyridine in the iodide complexes are less downfield shifted and are sharp, and of 2H intensity (in the spectrum of **4b–Rh–I**, all resonances are broad, not as a consequence of slowed pyridine rotation). The other (3-CH and 4-CH) pyridine resonances of the iodide complexes are very similar to each other in chemical shift, but not to those of the chloride complexes. Lastly, the pyrrolic resonances of the PAlP pincer ligand clearly show a pattern of different chemical shifts for the chloride complexes vs the iodide complexes.

The differences between the set of chloride complexes on one hand, and the iodide complexes on the other hand, are unlikely to be explained by an isostructural substitution of chloride with iodide. After all, the effect of the change from Rh to Ir, or from Me to H with the same halide is much less pronounced. We posit that the ^1^H NMR data, together with the X-ray structure of **3b–Rh–I**, support the notion that all the iodide complexes here are indeed **b**-type isomers, with a pyridine on Al and the iodide on the transition metal.

We also examined the ^31^P NMR data for the experimentally available compounds (Table S5). However, any patterns for the **a**/**b** isomer contrast were more difficult to discern.

The iodide complexes **3** and **4** proved to be poorly soluble in organic solvents of modest polarity (arenes, CH_2_Cl_2_), which led to difficulties in obtaining ^13^C NMR data. The compounds were somewhat more soluble in THF, which allowed us to collect ^13^C{^1^H} NMR spectra of **4b–Rh–I** and **4b–Ir–I**. However, dissolution of **3b–Rh–I** and **3b–Ir–I** in THF-*d*_8_ resulted in the observation of two resonances by ^31^P NMR and of free pyridine by ^1^H NMR spectroscopy, ostensibly indicating THF/pyridine exchange.

**Scheme 2:**
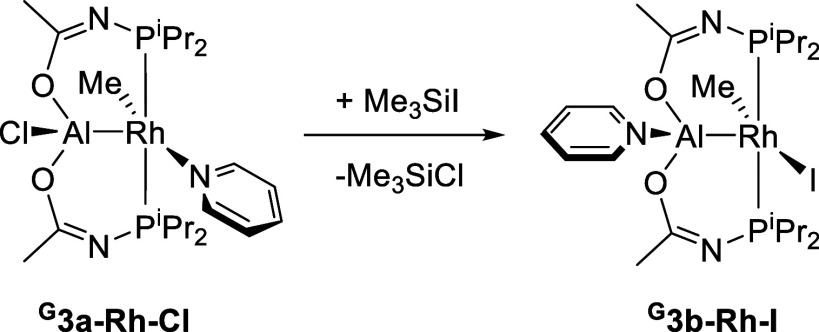
Synthesis of ^**G**^**3b–Rh–I**

Very recently, we disclosed the synthesis of Rh complexes of a closely related PAlP pincer **G** (Scheme 2), which was constructed with acetamide linkers in place of pyrrolic ones and resulted in two six-membered instead of five-membered metallacycles.^35^ With Me/py/Cl, the isomer we observed was ^**G**^**3a–Rh–Cl**. It displayed the splitting of the pyridine hydrogens into five inequivalent resonances and the telltale pattern of upfield-shifted P^i^Pr_2_ methyl groups by ^1^H NMR spectroscopy, similar to that discussed above for the compounds in this paper. The structure of ^**G**^**3a–Rh–Cl** was also confirmed by X-ray crystallography.^35^ Treatment of it with Me_3_SiI resulted in clean conversion to a new product, which we deem to be ^**G**^**3b–Rh–I**, based on the analysis of its NMR spectroscopic properties: its pyridine resonances are now in 2:2:1 ratio and none of the P^i^Pr_2_ methyl resonances are upfield of 1 ppm in the ^1^H NMR spectrum.

## Conclusions

In summary, we have examined the structural preferences in group 9 metal complexes supported by the PAlP pincer and also carrying a pyridine ligand, a halide, and a methyl or a hydride. DFT calculations predicted that a change in the nature of halide from chloride to iodide results in a different preferred isomer. With chloride, the transition metal binds pyridine and Al binds the halide, whereas with iodide, the transition metal binds iodide and Al binds pyridine. Experimental studies of the Rh and Ir complexes in solution by NMR spectroscopy, and in the solid state by X-ray crystallography, confirmed the computational predictions. This change in preference can be rationalized using the hard–soft acid–base concepts.

## Supporting Information Available

Details of experimental procedures and spectroscopic characterization (PDF)
Containing the coordinates of all the DFT-optimized structures (XYZ)

## Supplementary Material

om4c00490_si_001.pdf

om4c00490_si_002.xyz
