# Supplementary material for: Impact of Halide (Cl vs I) Identity on the Preferred Positioning of Substituents between Al and M (M = Co, Rh, Ir) in PAlP Pincer Complexes
Source: Organometallics. 2024 Dec 31;44(1):347–53. doi: 10.1021/acs.organomet.4c00490 (PMC11734121; doi:10.1021/acs.organomet.4c00490)
Supplement: Supplementary file 1 — om4c00490_si_001.pdf [file om4c00490_si_001.pdf]

Supporting Information

Impact of Halide (Cl vs I) Identity on the Preferred Positioning of Substituents between Al and M (M = Co, Rh, Ir) in PAlP Pincer Complexes

Samuel R. Lee [a], *Natchayatorn Keawkla* [b], *R. Noah Sladek* [a], *Nattamai Bhuvanesh* [a], *Panida Surawatanawong* [b],* *and Oleg V. Ozerov* [a]*

[a] Department of Chemistry, Texas A&M University, 3255 TAMU, College Station, TX 77842.

[b] Department of Chemistry and Center of Excellence for Innovation in Chemistry, Mahidol University, Bangkok 10400, Thailand.

ozerov@chem.tamu.edu, panida.sur@mahidol.ac.th

I. General Considerations **S3**
II. DFT computational details. **S4**
III. Synthesis of PAlP Metal Complexes **S11**
IV. Selected NMR Data Tables **S17**
V. NMR Spectra **S19**
VI. X-Ray Structural Determination Details **S47**
VII. SI References **S49**

I. General Considerations

Unless otherwise specified, all manipulations were performed either inside an argon-filled glove box, or by using Schlenk techniques. Pentane and toluene were dried using a PureSolv MD-5 Solvent Purification System and were stored over 4Å molecular sieves in an argon-filled glove box. Benzene (PhH), benzene-d_6 (C_6D_6), fluorobenzene (PhF), and pyridine were dried over CaH_2 and distilled, then stored in an argon-filled glovebox over 4Å molecular sieves prior to use. Iridium and Rhodium precursors $[(COD)IrCl]_2$,[1] $[(COD)RhCl]_2$,[2] and ligand **1**[3] were synthesized according to literature precedent. All other chemicals were used as received from commercial vendors. Argon was used from standard gas cylinders with 99.998% purity. NMR spectra were recorded on Bruker Avance Neo 400 (^{1}H NMR, 400.200 MHz; ^{13}C NMR, 100.630 MHz; ^{31}P NMR, 161.95 MHz), Inova 500 (^{1}H NMR, 499.431 MHz; ^{13}C NMR, 125.595 MHz; ^{31}P NMR, 202.187 MHz), and Avance Neo 500 (^{1}H NMR, 500.13 MHz; ^{13}C NMR, 125.77 MHz; ^{31}P NMR, 202.45 MHz) spectrometers. Chemical shifts are reported in δ (ppm). For ^{1}H and ^{13}C NMR spectra, the residual solvent peak was used as an internal reference (^{1}H NMR: δ 7.16 for C_6D_6, 3.58 and 1.72 for THF-d_8; ^{13}C NMR: δ 128.06 for C_6D_6, 67.21 and 25.31 for THF-d_8). ^{31}P NMR spectra were externally referenced to an 85% phosphoric acid solution δ 0. Elemental analyses were performed by Robertson Microlit Laboratories (Ledgewood, NJ).

II. DFT computational details.

Density functional calculations were carried out using the Guassian16 software package.[4] Geometries were optimized in gas-phase using M06[5] functional with SDD[6] basis set for Co, Rh, and Ir and 6-311G(d,p)[7–9] basis set for all other atoms. Frequency calculations were performed on the gas-phase optimized geometries to confirm that the located stationary points are minima and to obtain thermodynamic properties. The single-point calculations in solvent were performed on the gas-phase optimized geometries using SMD[10] continuum solvation model with solvent parameters for toluene (ε=2.3741). M06 functional with SDD basis was used for Co, Rh, and Ir and the 6-311+G(d,p)[7–9,11,12] basis set was used for all other atoms. Natural bond orbital (NBO) calculations were performed using NBO 6.0.[13] Cylview10[14] software was used to visualize optimized structure geometries.

Table S1. Natural Coulomb electrostatics (NCE) potential energy between fragment units in **3a** and **3b** complexes.

		unit 1	unit 2	NCE potential energy (kcal/mol)		unit 1	unit 2	NCE potential energy (kcal/mol)
Rh	**3a-Cl**	**Cl**	**Al**	**-151.1**	**3b-Cl**	pyridine	Al	-62.3
	**3a-I**	I	Al	-82.1	**3b-I**	pyridine	Al	-62.0

Table S2. Second-order perturbation energy ($E^{(2)}$) from donor to acceptor orbital interaction from NBO analysis.

		donor	acceptor	$E^{(2)}$ (kcal/mol)		donor	acceptor	$E^{(2)}$ (kcal/mol)
Rh	**3a-Cl**	LP(Cl)	LV(Al)	59.2	**3b-Cl**	LP(N)	LV(Al)	26.9
		LP(N)	LV(Rh)	57.7		LP(Cl)	LV(Rh)	54.2
	**3a-I**	LP(I)	LV(Al)	57.6	**3b-I**	LP(N)	LV(Al)	24.1
		LP(N)	LV(Rh)	57.3		**LP(I)**	**LV(Rh)**	**63.5**

LP = lone pair orbital; LV = lone vacant orbital

Table S3. Selected bond distances for **3a** and **4a** in Å and the relative free energy (ΔG(b-a)) in kcal/mol.

Bond distance (Å)	**Ir-Cl** **3a**	**Ir-Cl** **4a**	**Ir-I** **3a**	**Ir-I** **4a**	**Rh-Cl** **3a**	**Rh-Cl** **4a**	**Rh-I** **3a**	**Rh-I** **4a**	**Co-Cl** **3a**	**Co-Cl** **4a**	**Co-I** **3a**	**Co-I** **4a**
M-Al	2.395	2.393	2.398	2.393	2.383	2.381	2.386	2.381	2.295	2.299	2.297	2.302
M-R	2.121	1.600	2.122	1.600	2.108	1.585	2.110	1.586	1.988	1.500	1.990	1.498
M-N(py)	2.211	2.212	2.213	2.211	2.203	2.193	2.205	2.194	1.979	1.949	1.979	1.952
M-P1	2.365	2.344	2.368	2.346	2.367	2.343	2.370	2.345	2.266	2.219	2.270	2.220
M-P2	2.369	2.344	2.370	2.346	2.371	2.342	2.372	2.345	2.269	2.219	2.271	2.221
Al-X	2.178	2.177	2.602	2.597	2.176	2.176	2.600	2.596	2.187	2.183	2.619	2.605
M-X	-	-	-	-	-	-	-	-	-	-	-	-
Al-N(py)	-	-	-	-	-	-	-	-	-	-	-	-
Al--R(M)	2.972	2.344	2.937	2.282	2.882	2.138	2.850	2.109	2.663	1.904	2.616	1.894
X--(H-C2)py	2.606	2.810	2.919	3.113	2.628	2.865	2.947	3.125	2.590	2.701	2.896	3.009
**ΔG(b-a)**	7.3	7.7	-1.5	-7.8	6.9	6.5	-2.2	-4.4	4.3	4.8	-0.5	-3.4

***M = Ir, Rh, Co; X = Cl, I; R = H, Me**

Table S4. Selected bond distances for **3b** and **4b** in Å and the relative free energy (ΔG(b-a)) in kcal/mol.

Bond distance (Å)	**Ir-Cl** **3b**	**Ir-Cl** **4b**	**Ir-I** **3b**	**Ir-I** **4b**	**Rh-Cl** **3b**	**Rh-Cl** **4b**	**Rh-I** **3b**	**Rh-I** **4b**	**Co-Cl** **3b**	**Co-Cl** **4b**	**Co-I** **3b**	**Co-I** **4b**
M-Al	2.320	2.320	2.329	2.331	2.303	2.305	2.312	2.317	2.208	2.216	2.217	2.229
M-R	2.132	1.607	2.139	1.608	2.126	1.599	2.136	1.603	2.015	1.501	2.009	1.497
M-N(py)	-	-	-	-	-	-	-	-	-	-	-	-
M-P1	2.365	2.349	2.380	2.354	2.368	2.349	2.385	2.354	2.268	2.221	2.288	2.227
M-P2	2.364	2.350	2.372	2.354	2.368	2.349	2.380	2.353	2.266	2.222	2.280	2.228
Al-X	-	-	-	-	-	-	-	-	-	-	-	-
M-X	2.488	2.454	2.792	2.762	2.457	2.425	2.758	2.727	2.250	2.222	2.584	2.550
Al-N(py)	2.020	2.022	2.019	2.021	2.024	2.022	2.026	2.023	2.048	2.030	2.047	2.029
Al--R(M)	2.786	2.176	2.755	2.155	2.664	2.020	2.623	1.995	2.401	1.888	2.407	1.889
X--(H-C2)py	2.483	2.797	2.940	3.169	2.454	2.703	2.917	3.081	2.543	2.739	2.928	3.088
**ΔG(b-a)**	7.3	7.7	-1.5	-7.8	6.9	6.5	-2.2	-4.4	4.3	4.8	-0.5	-3.4

***M = Ir, Rh, Co; X = Cl, I; R = H, Me**

Table S5. The sum of selected bond distances for isomers **a** and **b** in Å.

Bond distance (Å)	**Ir-Cl**	**Ir-Cl**	**Ir-I**	**Ir-I**	**Rh-Cl**	**Rh-Cl**	**Rh-I**	**Rh-I**	**Co-Cl**	**Co-Cl**	**Co-I**	**Co-I**
	**3**	**4**	**3**	**4**	**3**	**4**	**3**	**4**	**3**	**4**	**3**	**4**
$(M\text{-}N + Al\text{-}X)_a$	4.389	4.389	4.815	4.808	4.379	4.369	4.805	4.790	4.166	4.132	4.598	4.557
$(M\text{-}X + Al\text{-}N)_b$	4.508	4.476	4.811	4.783	4.481	4.447	4.784	4.750	4.298	4.252	4.631	4.579
$(M\text{-}X + Al\text{-}N)_b$ - $(M\text{-}N + Al\text{-}X)_a$	0.119	0.087	-0.004	-0.025	0.102	0.078	-0.021	-0.040	0.132	0.120	0.033	0.022
ΔG(b-a)	7.3	7.7	-1.5	-7.8	6.9	6.5	-2.2	-4.4	4.3	4.8	-0.5	-3.4

***M = Ir, Rh, Co; X = Cl, I; R = H, Me**

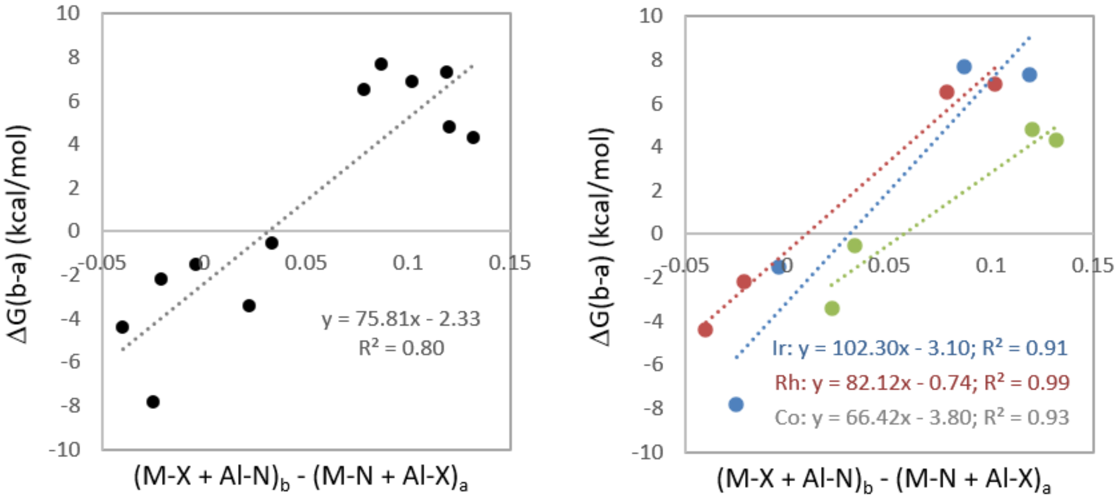

Figure S1. The correlation between the relative free energy (ΔG(b-a)) (in kcal/mol) and the difference in the sum of M-X and Al-N distances in isomer **b** and the sum of M-N and Al-X distances in isomer **a**, $(M\text{-}X + Al\text{-}N)_b$ - $(M\text{-}N + Al\text{-}X)_a$, shown in Å (M = Ir, Rh, Co; X = Cl, I).

Table S6. Natural population analysis (NPA) charges for **3a** and **4a**.

NPA charge	Ir-Cl	Ir-Cl	Ir-I	Ir-I	Rh-Cl	Rh-Cl	Rh-I	Rh-I	Co-Cl	Co-Cl	Co-I	Co-I
	3a	4a	3a	4a	3a	4a	3a	4a	3a	4a	3a	4a
M	-0.210	-0.400	-0.208	-0.398	-0.223	-0.397	-0.219	-0.390	-0.027	-0.237	-0.021	-0.231
Al	1.672	1.626	1.509	1.460	1.689	1.623	1.518	1.456	1.672	1.608	1.505	1.452
X	-0.590	-0.580	-0.432	-0.424	-0.587	-0.571	-0.424	-0.413	-0.584	-0.565	-0.425	-0.407
N(py)	-0.496	-0.519	-0.496	-0.521	-0.489	-0.506	-0.489	-0.507	-0.499	-0.504	-0.502	-0.506
ΔG(b-a)	7.3	7.7	-1.5	-7.8	6.9	6.5	-2.2	-4.4	4.3	4.8	-0.5	-3.4

***M = Ir, Rh, Co; X = Cl, I**

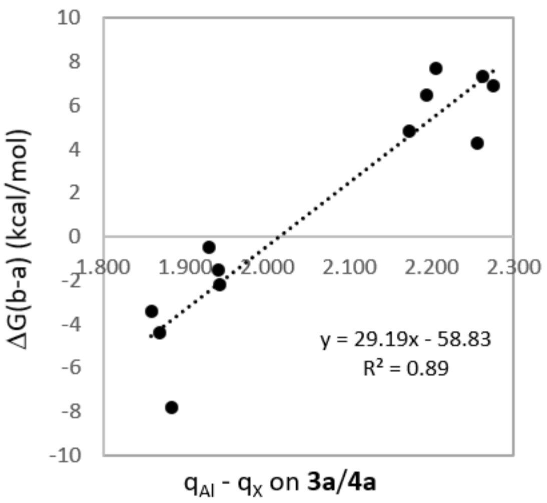

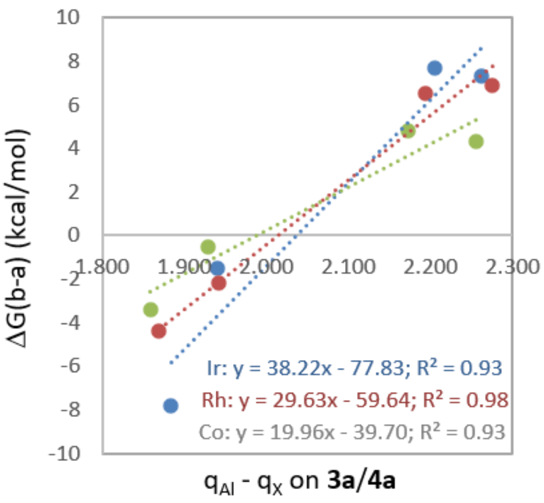

Figure S2. The correlation between the relative free energy (ΔG(b-a)) (in kcal/mol) and the difference in the NPA charge on Al and X (q_{Al} - q_X) (X = Cl, I) on **3a** and **4a**.

Table S7. Natural population analysis (NPA), Hirschfeld, and ESP charges for **3a-Rh-Cl**.

			NPA	Hirshfeld	ESP
Rh	**3a-Cl**	Rh	-0.223	-0.046	-0.547
		Al	1.689	0.349	0.698
		Cl	-0.587	-0.259	-0.435
		N(py)	-0.489	-0.059	0.391

Table S8. Maximum electrostatic potential (au) at the binding sites of halides and pyridine at Rh and Al.

		Maximum electrostatic potential (au)			Maximum electrostatic potential (au)	
		Al	Rh		Al	Rh
Rh	**3a-Cl**	0.609	0.160	**3b-Cl**	0.612	0.137
	**3a-I**	0.594	0.164	**3b-I**	0.616	0.137

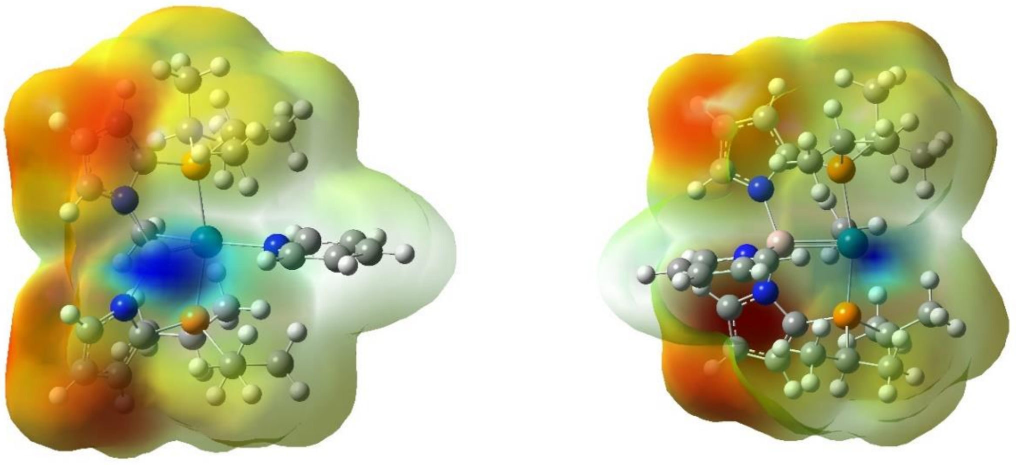

3a-Rh-Cl **3b-Rh-Cl**

Figure S3. Electrostatic potential plots (ESPs) mapped over electron density surfaces with isodensity of 0.02 au. The color scale is from 0.05 (red) to 0.19 (blue) au for (a) **3a-Rh-Cl** and (b) **3b-Rh-Cl**. The Cl atom is omitted for clarity.

Table S9. Selected bond distances for **3f** and **4f** in Å and the relative free energy (ΔG(f-a)) in kcal/mol.

Bond distance (Å)	Ir-Cl	Ir-I	Ir-Cl	Ir-I	Rh-Cl	Rh-I	Rh-Cl	Rh-I	Co-Cl	Co-I	Co-Cl	Co-I
	**4f**	**4f**	**3f**	**3f**	**4f**	**4f**	**3f**	**3f**	**4f**	**4f**	**3f**	**3f**
M-Al	2.478	2.480	2.490	2.495	2.480	2.480	2.492	2.500	2.432	2.441	2.462	2.470
M-R	-	-	-	-	-	-	-	-	-	-	-	-
M-N(py)	2.123	2.148	2.118	2.151	2.126	2.155	2.116	2.156	1.949	1.957	1.953	1.962
M-P1	2.391	2.398	2.395	2.399	2.394	2.401	2.400	2.402	2.302	2.306	2.305	2.310
M-P2	2.390	2.398	2.396	2.399	2.394	2.401	2.400	2.402	2.302	2.306	2.305	2.310
Al-X	-	-	-	-	-	-	-	-	-	-	-	-
M-X	2.372	2.687	2.372	2.686	2.345	2.657	2.346	2.655	2.204	2.55	2.203	2.548
Al-N(py)	-	-	-	-	-	-	-	-	-	-	-	-
Al-R	1.577	1.578	1.965	1.965	1.574	1.575	1.961	1.962	1.575	1.575	1.964	1.964
X--(H-C2)py	-	-	-	-	-	-	-	-	-	-	-	-
**ΔG(f-a)**	36.6	25.3	13.3	4.1	32.7	23.8	9.8	0.3	24.9	19.9	3.4	-2.6

***M = Ir, Rh, Co; X = Cl, I; R = H, Me**

Table S10. The sum of selected bond distances for isomers **f** and **a** in Å and relative free energy in kcal/mol

	**Bond distance (Å)**	**Ir-Cl**	**Ir-I**	**Ir-Cl**	**Ir-I**	**Rh-Cl**	**Rh-I**	**Rh-Cl**	**Rh-I**	**Co-Cl**	**Co-I**	**Co-Cl**	**Co-I**
		**4**	**4**	**3**	**3**	**4**	**4**	**3**	**3**	**4**	**4**	**3**	**3**
(1)	$(M\text{-}X + Al\text{-}R)_f$	3.949	4.265	4.337	4.651	3.919	4.232	4.307	4.617	3.779	4.125	4.167	4.512
(2)	$(M\text{-}R + Al\text{-}X)_a$	3.777	4.197	4.299	4.724	3.761	4.182	4.284	4.710	3.683	4.103	4.175	4.609
(3)	$(M\text{-}X + Al\text{-}R)_f - (M\text{-}R + Al\text{-}X)_a$	0.172	0.068	0.038	-0.073	0.158	0.050	0.023	-0.093	0.096	0.022	-0.008	-0.097
(4)	$(M\text{-}N)_f - (M\text{-}N)_a$	-0.089	-0.063	-0.093	-0.062	-0.067	-0.039	-0.087	-0.049	0.000	0.005	-0.026	-0.017
(5)	$(M\text{-}N + M\text{-}X + Al\text{-}R)_f - (M\text{-}N + M\text{-}R + Al\text{-}X)_a$	0.083	0.005	-0.055	-0.135	0.091	0.011	-0.064	-0.142	0.096	0.027	-0.034	-0.114
(6)	**ΔG(f-a)**	36.6	25.3	13.3	4.1	32.7	23.8	9.8	0.3	24.9	19.9	3.4	-2.6

***M = Ir, Rh, Co; X = Cl, I; R = H, Me**

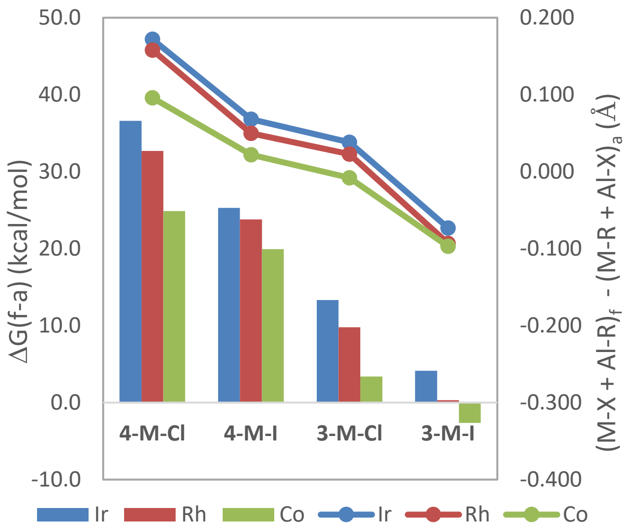

Figure S4. The relative free energy (ΔG(f-a)) (in kcal/mol) and the difference in the sum of M-X and Al-R distances in isomer **f** and the sum of M-R and Al-X distances in isomer **a**, (M-X + Al-R)$_f$ – (M-X + Al-R)$_a$, shown in Å for complexes **4-M-X** and **3-M-X** (M = Ir, Rh, Co; X = Cl, I; R = H, Me).

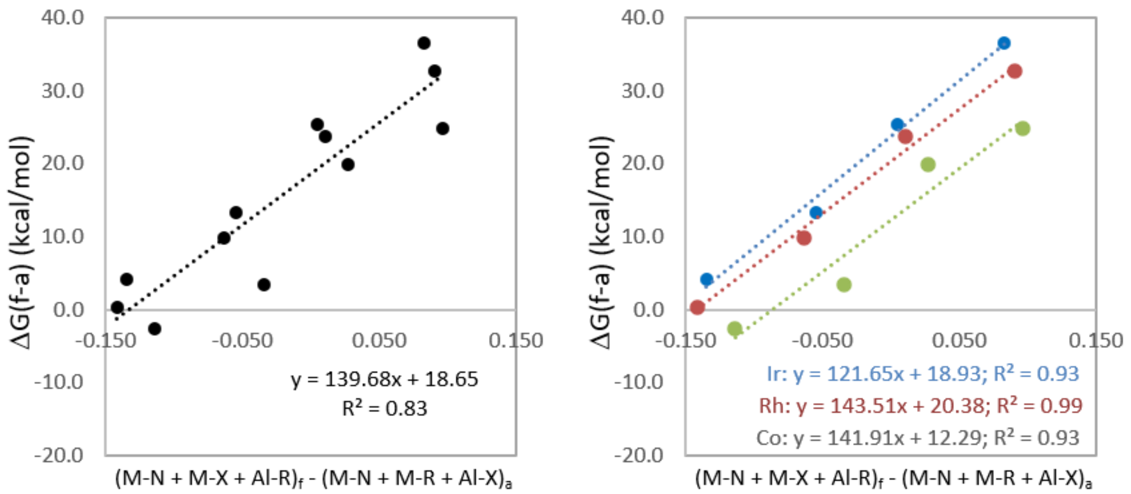

Figure S5. The correlation between the relative free energy (ΔG(f-a)) (in kcal/mol) and the difference in the sum of M-N, M-X, and Al-R distances in isomer **f** and the sum of M-N, M-R, and Al-X distances in isomer **a**, (M-N + M-X + Al-R)$_f$ - (M-N + M-X + Al-R)$_a$, shown in Å (M = Ir, Rh, Co; X = Cl, I; R = H, Me).

Table S11. Wiberg bond indices (WBIs) for **3a** and **4a** and the relative free energy (ΔG(d-a)) in kcal/mol.

	Ir-I	Ir-Cl	Ir-I	Ir-Cl	Rh-I	Rh-Cl	Rh-I	Rh-Cl	Co-I	Co-Cl	Co-I	Co-Cl
**WBI**	**4a**	**4a**	**3a**	**3a**	**4a**	**4a**	**3a**	**3a**	**4a**	**4a**	**3a**	**3a**
**Al--R(M)**	0.137	0.122	0.099	0.089	0.202	0.193	0.131	0.121	0.267	0.272	0.178	0.166
**ΔG(d-a)**	11.0	10.7	7.0	5.9	12.3	9.3	6.3	5.6	10.8	7.7	6.3	5.4

***M = Ir, Rh, Co; X = Cl, I; R = H, Me**

Table S12. Wiberg bond indices (WBIs) for **3b** and **4b** and the relative free energy (ΔG(e-b)) in kcal/mol.

	Ir-I	Ir-Cl	Ir-I	Ir-Cl	Rh-I	Rh-Cl	Rh-I	Rh-Cl	Co-I	Co-Cl	Co-I	Co-Cl
**WBI**	**4b**	**4b**	**3b**	**3b**	**4b**	**4b**	**3b**	**3b**	**4b**	**4b**	**3b**	**3b**
**Al--R(M)**	0.185	0.180	0.143	0.133	0.260	0.250	0.196	0.181	0.278	0.280	0.252	0.250
**ΔG(e-b)**	19.4	17.1	10.3	8.0	20.3	20.5	12.0	7.9	26.9	17.3	9.9	7.6

***M = Ir, Rh, Co; X = Cl, I; R = H, Me**

III. Synthesis of PAlP Metal Complexes

***In situ* generation of [(COD)RhI]$_2$.** A J. Young NMR tube was charged with 12 mg Me_3SiI (0.06 mmol) and 12 mg [(COD)RhCl]$_2$ (0.025 mmol) and dissolved in 0.6 mL C_6D_6 and the tube stirred for 30 minutes at room temperature to generate [(COD)RhI]$_2$ and Me_3SiCl, observable by ^{1}H NMR analysis. **[(COD)RhI]$_2$**: ^{1}H NMR (500 MHz, C_6D_6) δ 4.65 (brs, 4H), 1.97 (m, 4H), 1.22 (m, 4H).

***In situ* generation of [(COD)IrI]$_2$.** A J. Young NMR tube was charged with 12 mg Me_3SiI (0.06 mmol) and 17 mg [(COD)IrCl]$_2$ (0.025 mmol) dissolved in 0.6 mL C_6D_6 and the tube stirred for 30 minutes at room temperature to generate [(COD)IrI]$_2$ and Me_3SiCl, observable by ^{1}H NMR analysis. **[(COD)IrI]$_2$**: ^{1}H NMR (400 MHz, C_6D_6) δ 4.43 (m, 4H), 1.84 (m, 4H), 0.97 (m, 4H).

***In situ* generation of 2-Et.** A screw-capped test tube was charged with 37 mg **1** (0.20 mmol) and dissolved in 1 mL C_6D_6 before addition of 100 μL $AlEt_3$ (0.10 mmol, 1.0 M in heptane). The test

tube was placed in a 110 °C oil bath for 2 hours, and the solution analyzed by multinuclear NMR was estimated to contain 82% **2-Et**, 1% **2-H**,[15] and 17% unidentified byproducts. **2-Et:** ^{1}H NMR (400 MHz, C_6D_6) δ 7.31 (s, 2H, Pyrrole*H*), 6.75 (t, *J* = 2.4 Hz, 2H, Pyrrole*H*), 6.62 (d, *J* = 1.8 Hz, 2H, Pyrrole*H*), 1.97 (m, 4H, C*H*Me_2), 0.59 (m, 3H, $AlCH_2CH_3$), 0.44 (m, 2H, $AlCH_2CH_3$).

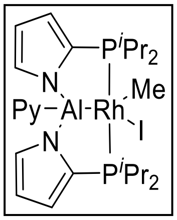

Synthesis of 3b-Rh-I. A 50 mL Schlenk flask was charged with 183 mg **1** (1.0 mmol) and 5 mL toluene before addition of 250 μL $AlMe_3$ (0.50 mmol, 2.0 M in toluene) with stirring for 3 hours to generate **2-Me**. Concurrently, a 25 mL Schlenk flask was charged with 123 mg $[(cod)RhCl]_2$ (0.25 mmol) and 2 mL CH_2Cl_2 before addition of 100 mg iodotrimethylsilane (0.50 mmol) with stirring for 3 hours to generate $[(COD)RhI]_2$. Volatiles were removed under vacuum. To the $[(COD)RhI]_2$ was added the toluene solution containing **2-Me** followed by 44 μL pyridine (0.55 mmol) with stirring for 20 hours, generating a bright yellow precipitate. The suspension was placed in a freezer at -35 °C for 1 hour and the yellow solid was collected by filtration over a medium frit. The solid was washed with cold toluene to give 253 mg **3b-Rh-I** (71%). A screw-capped test tube was charged with 30 mg **3b-Rh-I** suspended in 3 mL PhF and the tube placed in a 100 °C oil bath for 4 hours to afford a homogeneous solution. The tube was removed from the oil bath and allowed to cool to room temperature overnight, affording crystals suitable for X-ray diffraction studies. When dissolved in THF-d_8, free pyridine was observed alongside a new product in 75% abundance, tentatively assigned as the product of Al–Py displacement by THF-d_8 due to observation of free pyridine alongside apparently overlapping PAlP signals in the ^{1}H NMR spectrum. ^{1}H NMR (400 MHz, C_6D_6) δ 8.26 (m, 2H, Py*H*), 7.07 (m, 2H, Pyrrole*H*), 6.76 (m, 2H, Pyrrole*H*), 6.70 (t, *J* = 2.7 Hz, 2H, Pyrrole*H*), 6.44 (tt, *J* = 7.7, 1.5 Hz, 1H, Py*H*), 6.11 (m, 2H, Py*H*), 3.10 (m, 2H, C*H*Me_2), 2.52

(m, 2H, C*H*Me$_2$), 1.45 (dvt, $J_{HH} = J_{HP}$ = 7.0 Hz, 6H, CH*Me$_2$*), 1.39 (dvt, $J_{HH} = J_{HP}$ = 7.5 Hz, 6H, CH*Me$_2$*), 1.34 – 1.18 (m, 12H, CH*Me$_2$*), 0.72 (td, J_{HP} = 6.2 Hz, J_{HRh} = 1.4 Hz, 3H, Rh–*Me* ^{31}P{^{1}H} NMR (162 MHz, C_6D_6): 34.4 (d, J_{PRh} = 113 Hz). ^{31}P{^{1}H} NMR (162 MHz, THF-*d$_8$*): 34.4 (d, J_{RhP} = 113 Hz), 33.6 (d, J_{PRh} = 113 Hz).

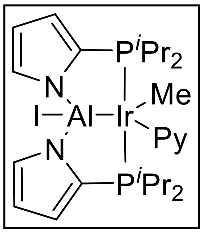

Synthesis of 3b-Ir-I. A 20 mL scintillation vial was charged with 168 mg [(cod)IrCl]$_2$ (0.25 mmol) and 2 mL toluene before addition of 100 mg iodotrimethylsilane (0.50 mmol) with stirring for 1 hour to generate [(COD)IrI]$_2$. Volatiles were removed under vacuum. Separately, a screw-capped test tube was charged with 183 mg **1** (1.0 mmol) and 5 mL toluene before addition of 250 μL AlMe$_3$ (0.50 mmol, 2.0 M in toluene) with stirring for 3 hours to generate **2-Me**. This solution was added to the vial containing [(COD)IrI]$_2$ along with 44 μL pyridine (0.55 mmol) with stirring for 2 hours, generating a bright yellow precipitate. The solution was placed in a -35 °C freezer overnight to give yellow crystals which were collected over a medium frit and washed with pentane (4 x 2 mL) to give 196 mg **3b-Ir-I** (49%). When dissolved in THF-*d$_8$*, solubility remained poor, and free pyridine was observed alongside a new product in 50% abundance, tentatively assigned as the product of Al–Py displacement by THF-*d$_8$* due to observation of free pyridine alongside apparently overlapping PAlP signals in the ^{1}H NMR spectrum. ^{1}H NMR (400 MHz, C_6D_6) δ 8.10 (dt, *J* = 5.1, 1.6 Hz, 2H, Py*H*), 7.05 (m, 2H, Pyrrole*H*), 6.78 (dd, *J* = 3.2, 1.0 Hz, 2H, Pyrrole*H*), 6.74 (ddt, *J* = 3.1, 2.2, 1.0 Hz, 2H, Pyrrole*H*), 6.44 (tt, *J* = 7.7, 1.6 Hz, 1H, Py*H*), 6.07 (m, 2H, Py*H*), 3.43 (m, 2H, C*H*Me$_2$), 2.78 (m, 2H, C*H*Me$_2$), 1.46 (dvt, $J_{HH} = J_{HP}$ = 7.1 Hz, 6H, CH*Me$_2$*), 1.38 (dvt, $J_{HH} = J_{HP}$ = 7.4 Hz, 6H, CH*Me$_2$*), 1.31 – 1.19 (m, 12H, CH*Me$_2$*), 0.98 (t, J_{HP} = 6.1 Hz, 3H, Ir–*Me*). ^{31}P{^{1}H} NMR

(202 MHz, C_6D_6): δ 31.0. $^{31}P\{^1H\}$ NMR (162 MHz, THF-d_8): δ 31.0, 30.8. Elem. Anal. Calc'd for (**3-Ir-I)$_3$·Toluene** C, 40.74; H, 5.39; N, 5.03. Found: C, 41.02; H, 5.63; N, 4.69.

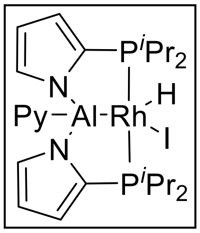

Synthesis of 4b-Rh-I. A screw-capped test tube was charged with stirbar and 100 mg Me_3SiI (0.50 mmol) dissolved in 2 mL toluene to which 123 mg $[(COD)RhCl]_2$ (0.25 mmol) was added with stirring for 30 minutes to generate $[(COD)RhI]_2$. Volatiles were removed under vacuum. Separately, 183 mg **1** (1.0 mmol) and 500 µL $AlEt_3$ (0.50 mmol, 1.0M in heptane) were mixed in a screw-cap test tube, dissolved in 5 mL toluene, and heated in a 110 °C bath for 2 hours to generate **2-Et**. The solution containing **2-Et** was added to the powdered $[(COD)RhI]_2$ before addition of 5 mL toluene (total volume 10 mL) with 44 µL pyridine (0.55 mmol). Upon addition of pyridine, yellow precipitate began forming rapidly. The suspension was then placed in a 50 °C oil bath for 1 hour. The suspension was placed in a -35 °C freezer overnight, and the precipitate collected on a frit and washed with cold pentane (3 x 2 mL) to give 256 mg of **4b-Rh-I** as a fluffy, pale-yellow powder (73%). ^1H NMR (400 MHz, C_6D_6) δ 8.08 (brs, 2H, Py*H*), 7.06 (m, 2H, Pyrrole*H*), 6.77 (t, J = 2.8 Hz, 2H, Pyrrole*H*), 6.72 (d, J = 2.8 Hz, 2H, Pyrrole*H*), 6.40 (brs, 1H, Py*H*), 6.05 (brs, 2H, Py*H*), 3.22 (m, 2H, C*H*Me_2), 2.34 (m, 2H, C*H*Me_2), 1.40 (m, 6H, CH*Me_2*), 1.24 (dvt, $J_{HH} = J_{HP}$ = 6.6 Hz, 6H, CH*Me_2*), 1.16-1.06 (m, 12H, CH*Me_2*), -16.18 (dt, J_{HRh} = 33.3 Hz, J_{HP} =16.3 Hz, 1H, Rh–*H*). ^1H NMR (500 MHz, THF-d_8) δ 8.47 (d, J = 5.3 Hz, 2H, Py*H*), 7.96 (brs, 1H, Py*H*), 7.49 (brs, 2H, Py*H*), 7.19 (m, 2H, Pyrrole*H*), 6.59 (d, J = 3.2 Hz, 2H, Pyrrole*H*), 6.43 (m, 2H, Pyrrole*H*), 2.99 (m, 2H, C*H*Me_2), 2.48 (m, 2H, C*H*Me_2), 1.31 (m, 12H, CH*Me_2*), 0.99 (dvt, $J_{HH} = J_{HP}$ = 7.6 Hz, 12H, CH*Me_2*), -16.71 (dd, J_{HRh} = 33.1, J_{HP} = 16.8 Hz, 1H, Rh–*H*). $^{31}P\{^1H\}$ NMR (202 MHz, C_6D_6): δ 38.6 (d, J_{HRh} = 112 Hz). $^{31}P\{^1H\}$ NMR (202 MHz, THF-d_8) δ 38.4 (d, J_{RhP} = 112 Hz). $^{13}C\{^1H\}$ NMR (126 MHz, THF-d_8): δ 149.5 (brs, Py*C*),

132.8 (t, J_{CP} = 31 Hz, Pyrrole*C*), 127.3 (t, J_{CP} = 6.1 Hz, Pyrrole*C*), 125.4 (brs, Py*C*), 116.2 (Pyrrole*C*), 114.1 (Pyrrole*C*), 28.4 (t, J_{CP} = 14.7 Hz, *C*HMe$_2$), 26.3 (t, J_{CP} = 13.3 Hz, *C*HMe$_2$), 21.0 (CH*Me$_2$*), 20.1 (CH*Me$_2$*), 19.6 (t, J_{CP} = 4.1 Hz, CH*Me$_2$*), 17.4 (CH*Me$_2$*).

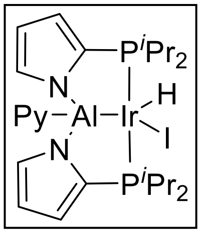

Synthesis of 4b-Ir-I. In a 20 mL scintillation vial, 168 mg [(cod)IrCl]$_2$ (0.25 mmol) and 1000 mg TMSI (0.50 mmol) were mixed in toluene, stirring at ambient temperature for 1 hour to generate [(COD)IrI]$_2$ before volatiles were removed under vacuum. Separately, 183 mg **1** (1.0 mmol) and 500 uL AlEt$_3$ (0.50 mmol, 1.0 M in heptane) were mixed in a screw-cap test tube, dissolved in 5 mL toluene, and heated in a 110 °C bath for 2 hours to generate **2-Et**. The [(COD)IrI]$_2$ was added to the solution containing **2-Et** with 44 μL pyridine (0.55 mmol) and 5 mL toluene (total volume 10 mL) before heating at 110 °C for 2 hours to give an orange solution. The vial was placed in a -35 °C freezer overnight to give yellow crystals. The crystals were collected on a frit and washed with pentane (4 x 2 mL) to give 166 mg **4b-Ir-I** (42%). ^{1}H NMR (400 MHz, C$_6$D$_6$) δ 7.95 (d, *J* = 5.6 Hz, 2H, Py*H*), 7.04 (d, *J* = 10.5 Hz, 2H, Pyrrole*H*), 6.82 (s, 2H, Pyrrole*H*), 6.74 (d, *J* = 3.3 Hz, 2H, Pyrrole*H*), 6.42 (t, *J* = 7.9 Hz, 1H, Py*H*), 6.02 (t, *J* = 6.7 Hz, 2H, Py*H*), 3.39 (p, *J* = 7.0 Hz, 2H), 2.71 (d, *J* = 9.0 Hz, 2H), 1.34 (dvt, $J_{HH} = J_{HP}$ = 7.4 Hz, 6H, CH*Me$_2$*), 1.27 (dvt, $J_{HH} = J_{HP}$ = 6.6 Hz, 6H, CH*Me$_2$*) 1.14 (dvt, $J_{HH} = J_{HP}$ = 7.3 Hz, 6H, CH*Me$_2$*), 1.07 (dvt, $J_{HH} = J_{HP}$ = 7.6 Hz, 6H, CH*Me$_2$*), -19.94 (t, J_{HP} = 15.3 Hz, 1H, Ir–*H*). ^{1}H NMR (500 MHz, THF-*d$_8$*) δ 8.39 (brs, 2H, Py*H*), 8.00 (brs, 1H, Py*H*), 7.51 (brs, 2H, Py*H*), 7.16 (s, 2H, Pyrrole*H*), 6.57 (s, 2H, Pyrrole*H*), 6.46 (s, 2H, Pyrrole*H*), 3.13 (m, 2H, C*H*Me$_2$), 2.82 (brs, 2H, C*H*Me$_2$), 1.32 (dvt, $J_{HH} = J_{HP}$ = 6.7 Hz, 6H, CH*Me$_2$*), 1.24 (dvt, $J_{HH} = J_{HP}$ = 7.2 Hz, 6H, CH*Me$_2$*), 1.07 – 0.74 (m, 12H, CH*Me$_2$*), -20.53 (t, J_{HP} = 15.3 Hz, 1H, Ir–*H*). ^{31}P{^{1}H} NMR (162 MHz, C$_6$D$_6$): δ 39.5. ^{31}P{^{1}H} NMR (202 MHz, THF-*d$_8$*) δ 39.3. ^{13}C{^{1}H} NMR (101 MHz, THF-

d$_8$): δ 149.3 (brs, Py*C*), 132.8 (t, J_{CP} = 35.5 Hz, Pyrrole*C*), 126.8 (t, J_{CP} = 5.8 Hz, Pyrrole*C*), 125.9 (brs, Py*C*), 115.5 (t, J_{CP} = 4.1 Hz, Pyrrole*C*), 113.8 (t, J_{CP} = 2.9 Hz, Pyrrole*C*), 28.1 (t, J_{CP} = 16.8 Hz, *C*HMe$_2$), 25.8 (t, J_{CP} = 15.7 Hz, *C*HMe$_2$), 21.0 (CH*Me*$_2$), 19.7 (CH*Me*$_2$), 19.0 (t, J_{CP} = 4.0 Hz, CH*Me*$_2$), 17.5 (CH*Me*$_2$). Elem. Anal. Calc'd for **(4-Ir-I)$_3$·Toluene** C, 39.97; H, 5.24; N, 5.12. Found: C, 39.97; H, 5.25; N, 4.75.

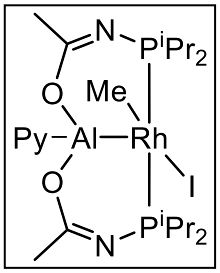

***In-situ* observation of ^{G}3b-Rh-I.** Me$_3$SiI (5.5 µL, 0.038 mmol) was added to a solution of **^{G}3a-Rh-Cl** (20 mg, 0.038 mmol) in 600 µL C_6D_6 in a J. Young tube and stirred at room temperature for 1 h. NMR analysis of the mixture revealed formation of **^{G}3b-Rh-I** (and Me$_3$SiCl). ^{1}H NMR (400 MHz, C_6D_6): δ 9.28 (dt, $J_{H–H}$ = 5.2 Hz, $J_{H–H}$ = 1.6 Hz, 2H, Py*H*), 6.71 (tt, *J* = 7.7, 1.7 Hz, 1H, Py*H*), 6.45 (m, 2H, Py*H*), 3.24 (hept, *J* = 3.2 Hz, 2H, C*H*Me$_2$), 2.48 (m, 2H, C*H*Me$_2$), 2.10 (s, 6H, *Me*C(=N)O), 1.53 (m, 12H, CH*Me*$_2$), 1.21 (dvt, J_{HH} = J_{HP} = 6.3 Hz, 6H, CH*Me*$_2$), 1.11 (dvt, J_{HH} = J_{HP} = 7.5 Hz, 6H, CH*Me*$_2$), 0.80 (td, J_{HP} = 6.1 Hz, J_{HRh} = 1.4 Hz, 3H, Rh–*Me*). ^{31}P{^{1}H} NMR (162 MHz, C_6D_6): δ 71.7 (d, J_{RhP} = 104.3 Hz). ^{13}C{^{1}H} NMR (101 MHz, C_6D_6): δ 170.0 (s, Me*C*(=N)O), 149.0 (s, Py*C*), 141.3 (s, Py*C*), 124.6 (s, Py*C*), 30.4 (t, $J_{P–C}$ = 16.5 Hz, *C*HMe$_2$), 29.8 (t, J_{CP}= 4.8 Hz, *C*HMe$_2$), 26.7 (t, J_{CP}= 4.8 Hz, CH*Me*$_2$), 20.1 (s, *Me*C(=N)O), 19.8 (t, J_{CP}= 3.5 Hz, CH*Me*$_2$), 19.7 (s, CH*Me*$_2$), 18.0 (s, CH*Me*$_2$). *Rh–Me not observed, presumably due to low signal/noise ratio for the expected doublet of triplets.*

IV. Selected NMR Data Tables

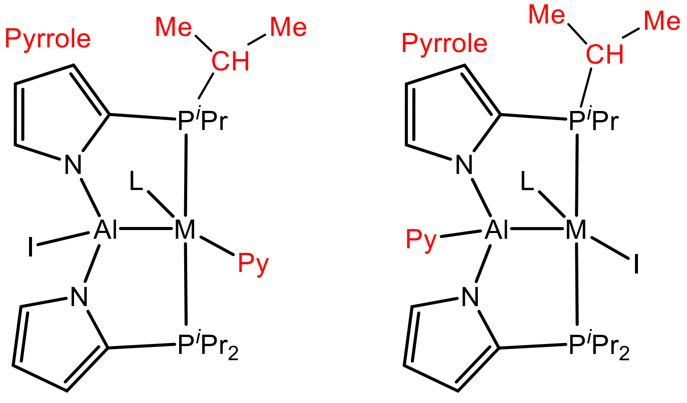

(3/4)a-M-I **(3/4)b-M-I**

Table S13. Comparison of ^{1}H NMR signals in the aromatic region in various solvents for compounds **3-** and **4-M-I**.

Compound	Solvent	Pyrrole*H**			Py*H**		
**3b-Rh-I#**	C_6D_6	7.07	6.76	6.70	8.26	6.44	6.11
	THF-d_8		-			-	
**3b-Ir-I#**	C_6D_6	7.05	6.78	6.74	8.10	6.44	6.07
	THF-d_8		-			-	
**4b-Rh-I**	C_6D_6	7.06	6.77	6.72	8.08	6.40	6.05
	THF-d_8	7.19	6.59	6.43	8.47	7.96	7.49
**4b-Ir-I**	C_6D_6	7.04	6.82	6.74	7.95	6.42	6.02
	THF-d_8	7.16	6.57	6.46	8.39	8.00	7.51

*****Signal intensities of 2:2:2 for Pyrrole*H* and 2:1:2 for Py*H*.

#Signals for **3** series overlap with Al–*Py* displacement product in THF–d_8.

Table S14. Comparison of ^{1}H NMR signals in the aliphatic and M–L regions in various solvents for compounds **3-** and **4-M-I**.

Compound	Solvent	C*H*		*Me*				M-*L**
**3b-Rh-I**#	C_6D_6	3.10	2.52	1.45	1.39	1.34-1.18		0.72
	THF-d_8	-		-				-
**3b-Ir-I**#	C_6D_6	3.43	2.78	1.46	1.38	1.31-1.19		0.98
	THF-d_8	-		-				-
**4b-Rh-I**	C_6D_6	3.22	2.35	1.40	1.24	1.11		-16.14
	THF-d_8	3.00	2.48	1.30		0.98		-16.73
**4b-Ir-I**	C_6D_6	3.39	2.71	1.34	1.27	1.14	1.07	-19.94
	THF-d_8	3.13	2.82	1.32	1.23	1.07-0.74		-20.51

***L** = Me for **3-** and H for **4-** series compounds.

#Signals for **3** series overlap with Al–*Py* displacement product in THF–d_8.

Table S15. Comparison of $^{31}P\{^{1}H\}$ NMR signals in **3-** and **4-** series compounds. Reported in δ, ppm (J_{RhP}, Hz) with C_6D_6 as solvent.

Compound	3b-Rh-I	3b-Ir-I	4b-Rh-I	4b-Ir-I	3a-Rh-Cl	3a-Ir-Cl	4a-Rh-Cl	4a-Ir-Cl
^{31}P	34.4 (113)	31.0	38.6 (112)	39.5	33.7 (120)	31.1	34.2 (121)	36.3

Rh-Cl and **Ir-Cl** complex data taken from SI reference 15.

V. NMR Spectra

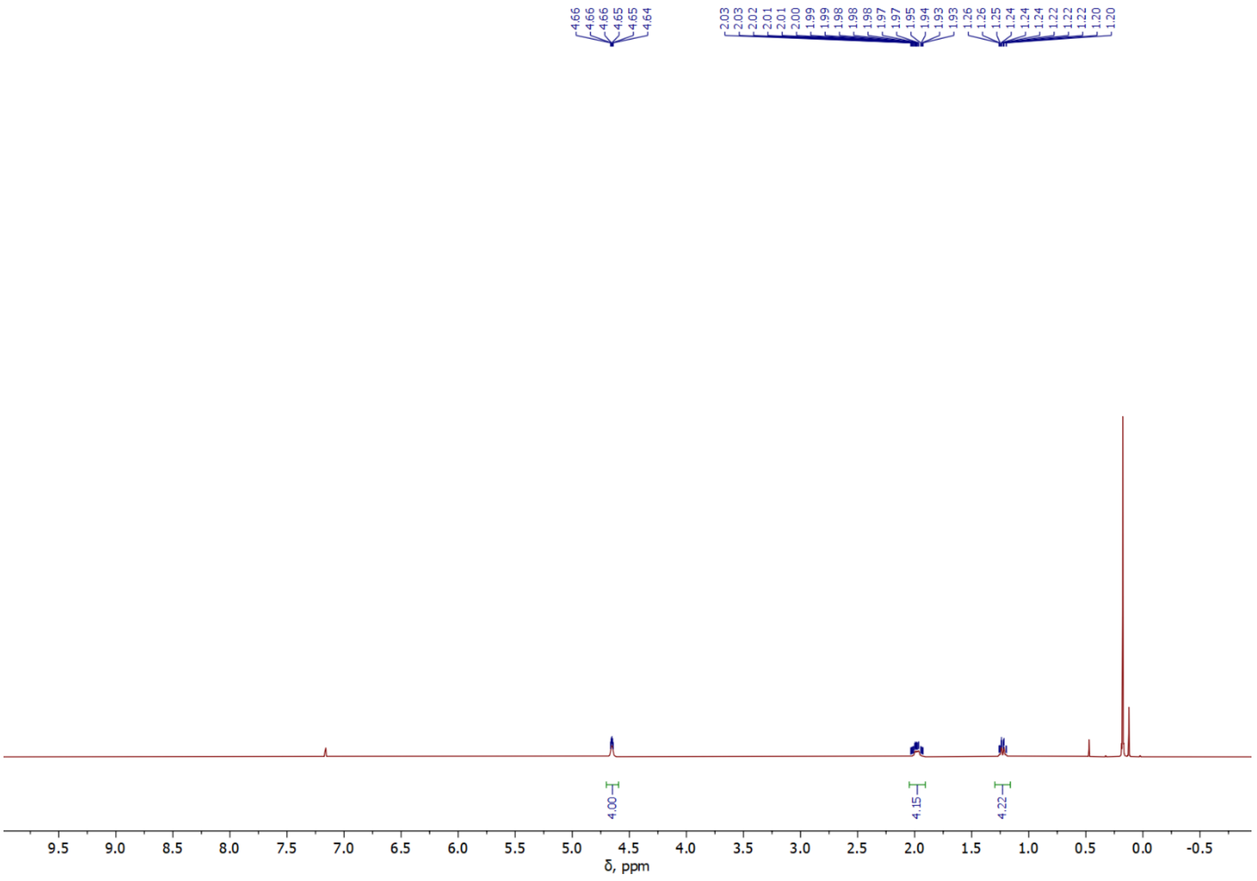

Figure S6. ^{1}H NMR (400 MHz, C_6D_6) of the *in situ* generation of **[(COD)RhI]$_2$** and Me_3SiCl (δ 0.18 ppm).

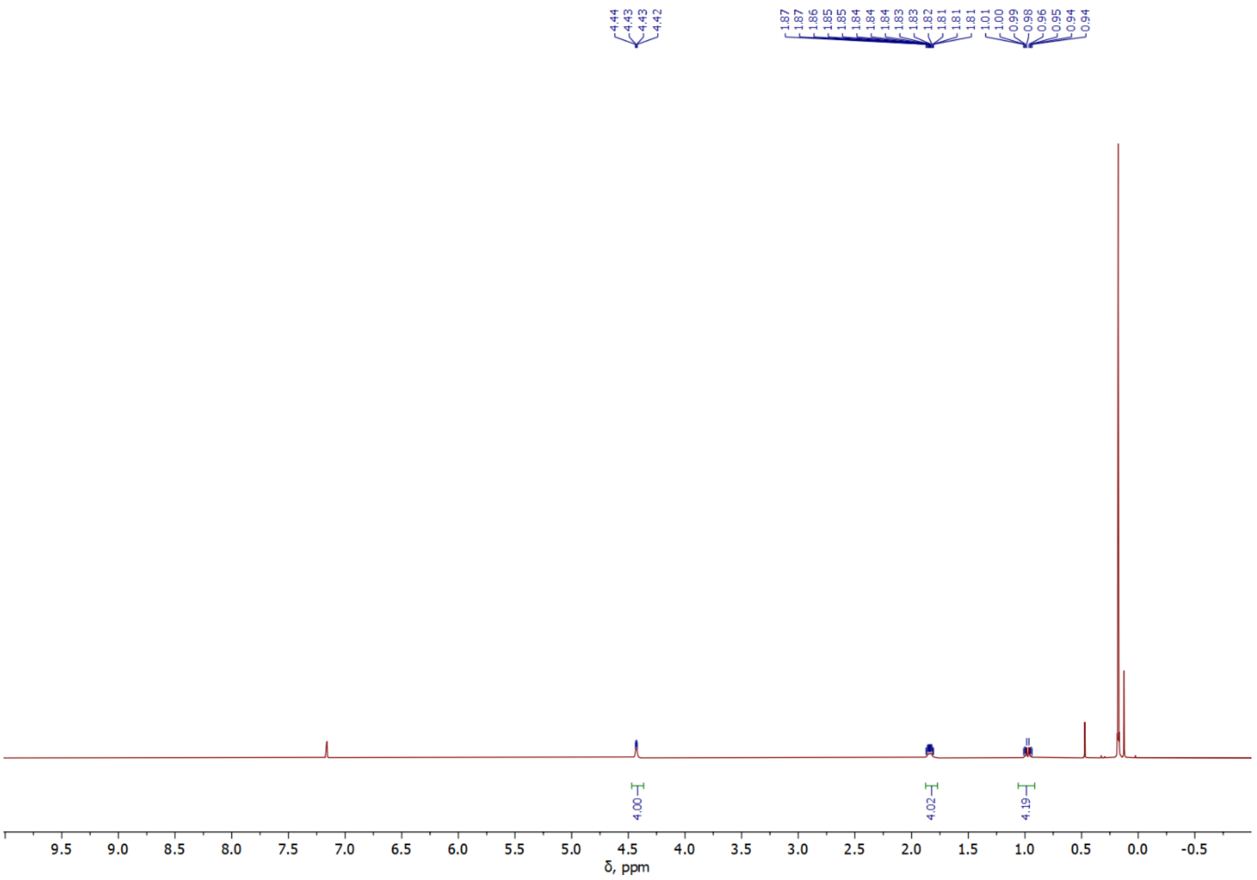

Figure S7. ^{1}H NMR (400 MHz, C_6D_6) of the *in situ* generation of **$[(COD)IrI]_2$** and Me_3SiCl (δ 0.18 ppm).

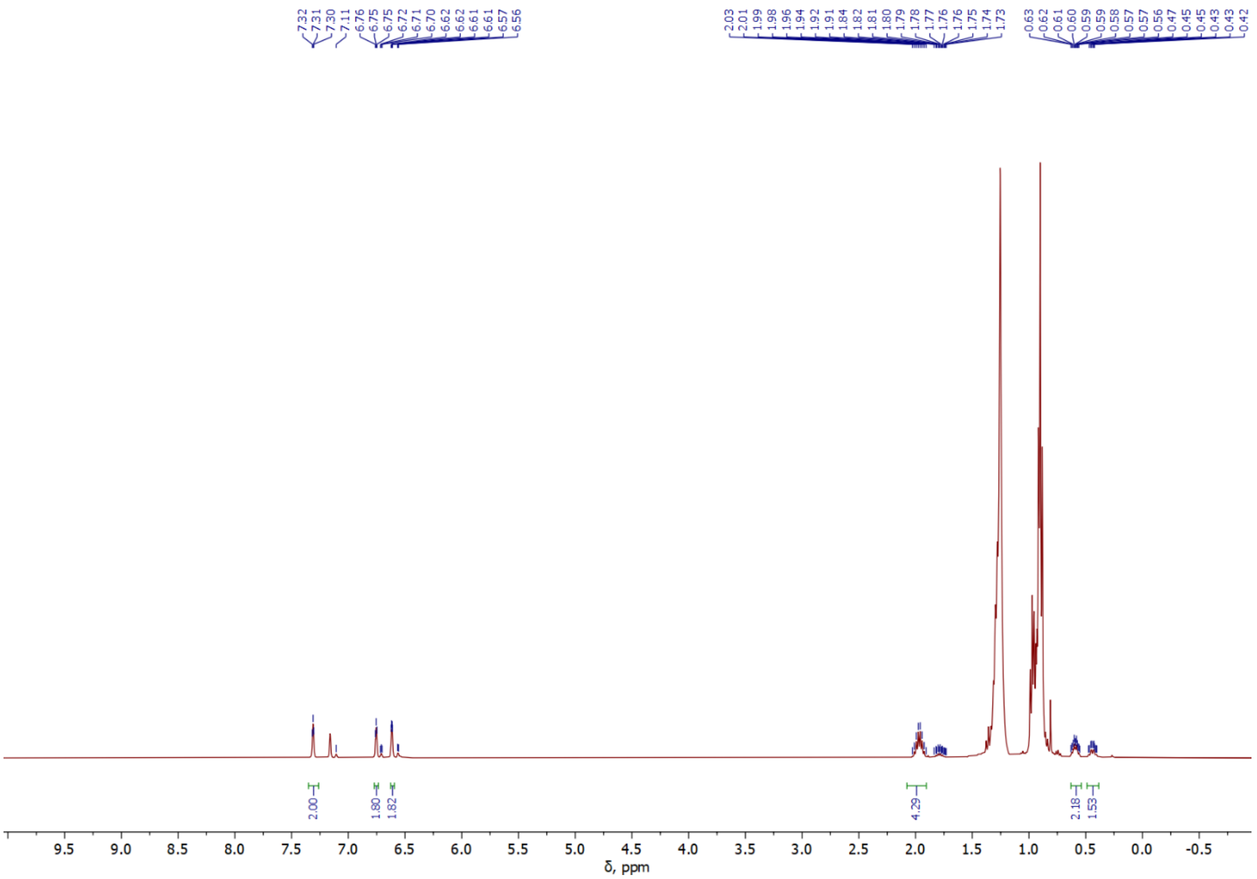

Figure S8. ^{1}H NMR (400 MHz, C_6D_6) of the *in situ* generation of **2-Et** (82%).

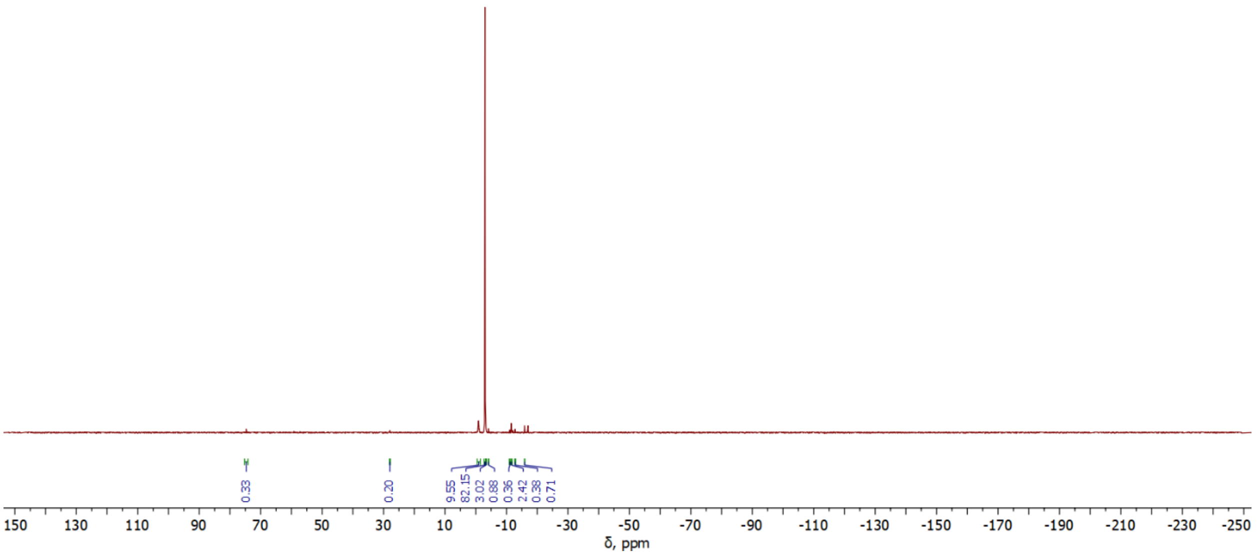

Figure S9. $^{31}P\{^{1}H\}$ NMR (162 MHz, C_6D_6) of the *in situ* generation of **2-Et** (82%).

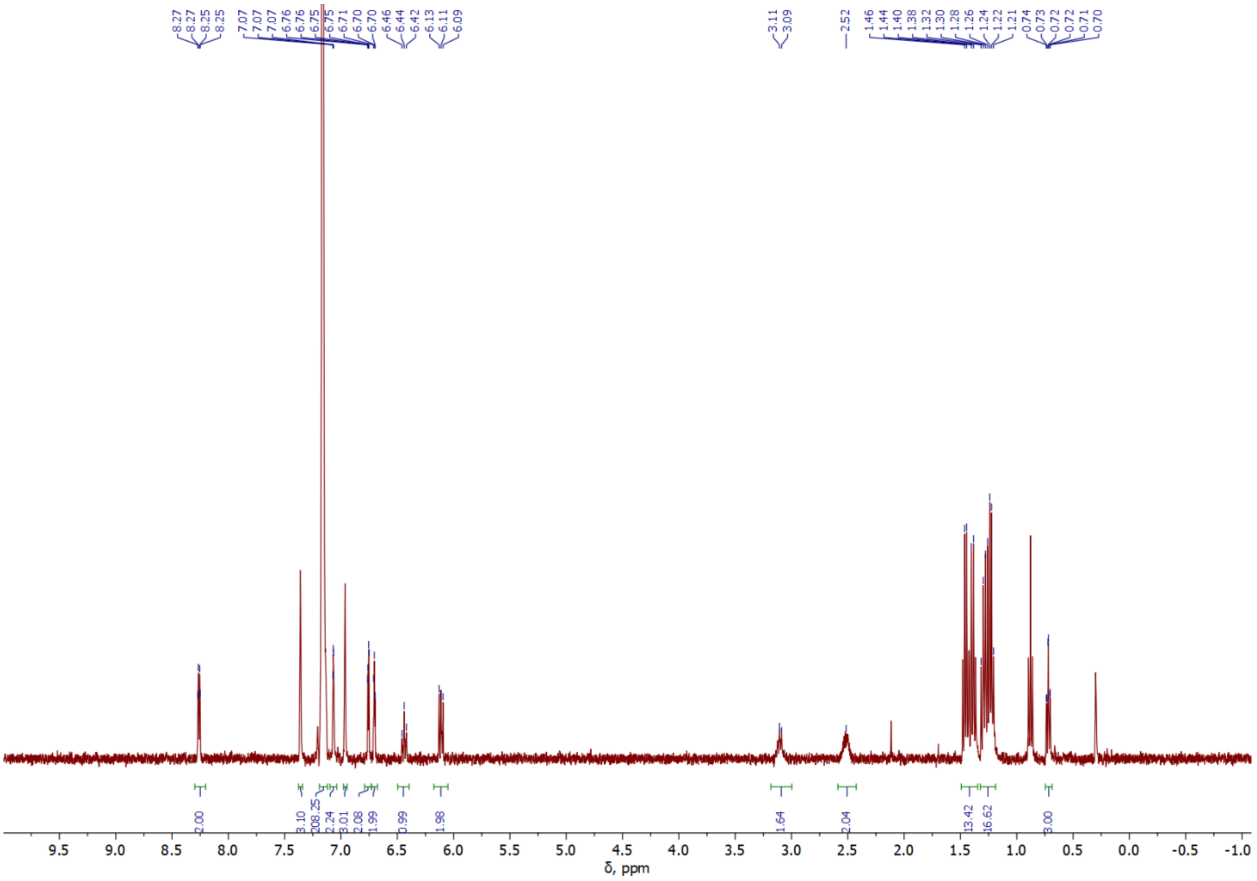

Figure S10. ^{1}H NMR (400 MHz, C_6D_6) of **3b-Rh-I**, extremely poor solubility.

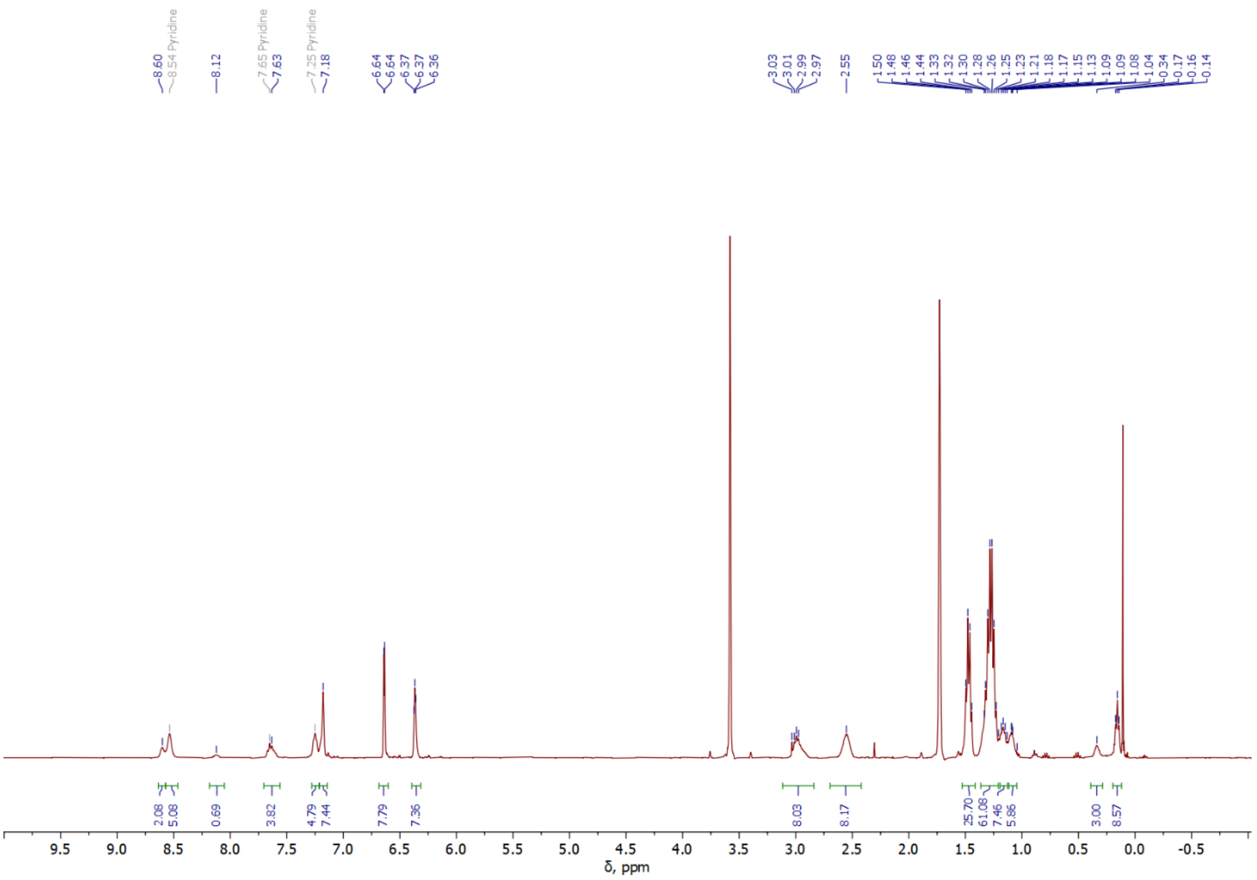

Figure S11. ^{1}H NMR (400 MHz, THF-d_8) upon dissolution of **3b-Rh-I** in THF-d_8.

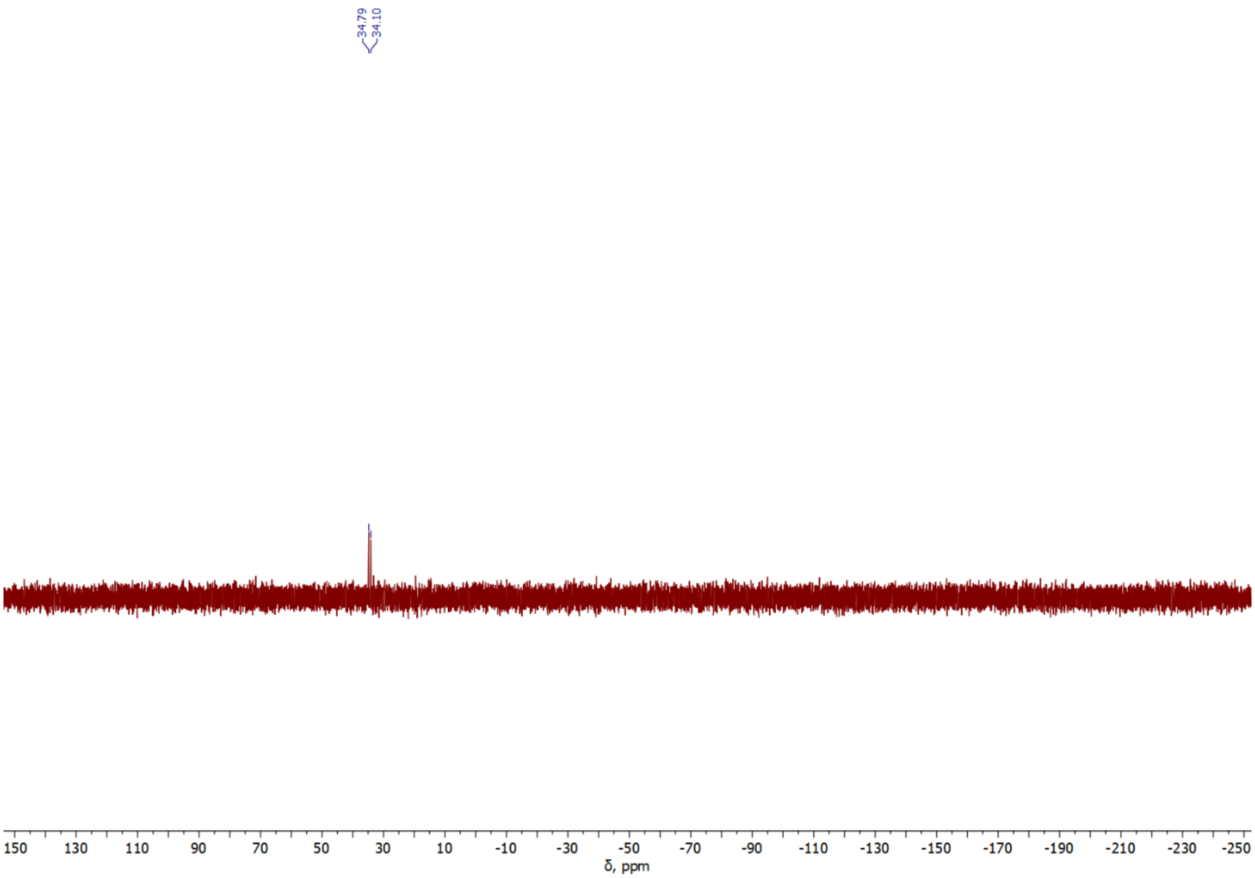

Figure S12. $^{31}P\{^{1}H\}$ NMR (162 MHz, C_6D_6) of **3b-Rh-I**, extremely poor solubility.

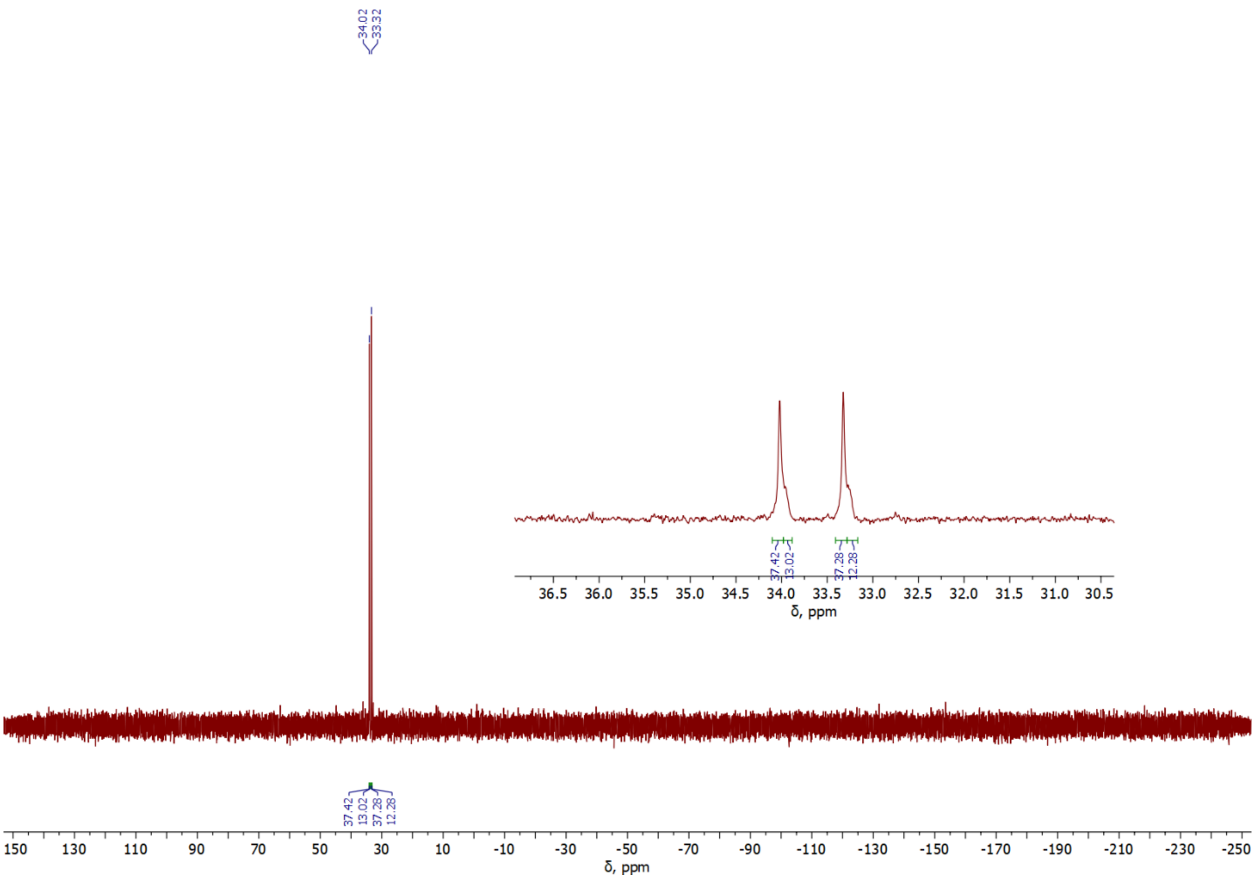

Figure S13. $^{31}P\{^{1}H\}$ NMR (162 MHz, THF-d_8) upon dissolution of **3b-Rh-I** in THF-d_8.

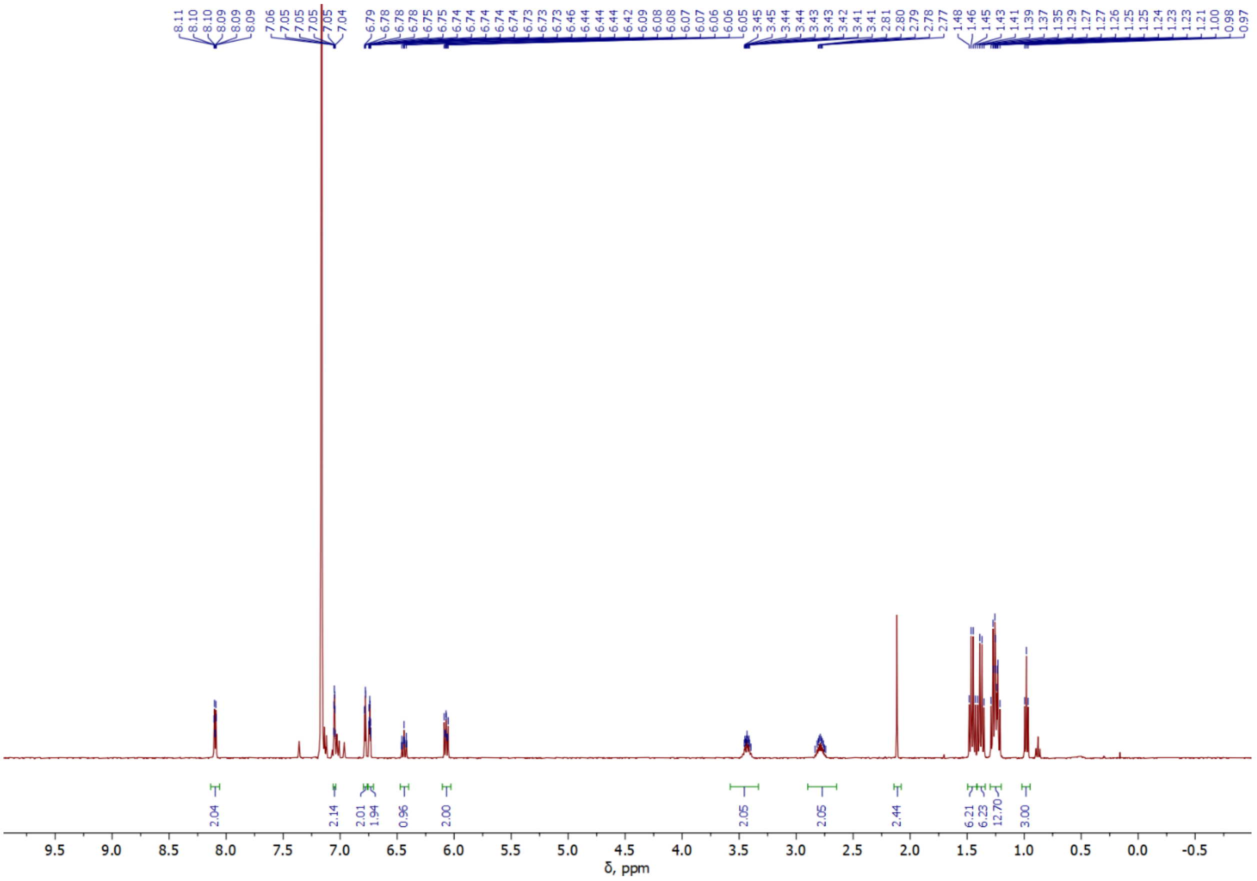

Figure S14. ^{1}H NMR (400 MHz, C_6D_6) of **3b-Ir-I**.

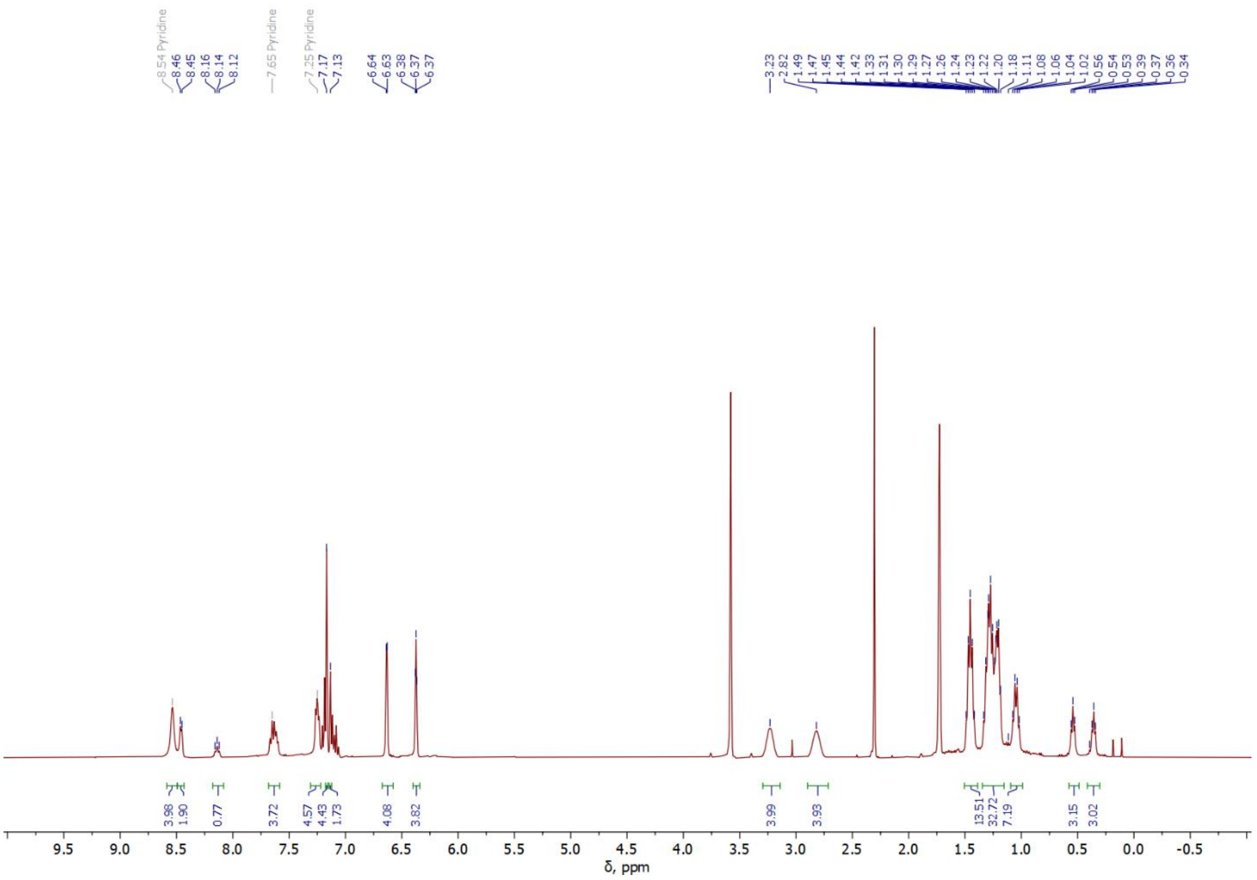

Figure S15. ^{1}H NMR (400 MHz, THF-d_8) upon dissolution of **3b-Ir-I** in THF-d_8.

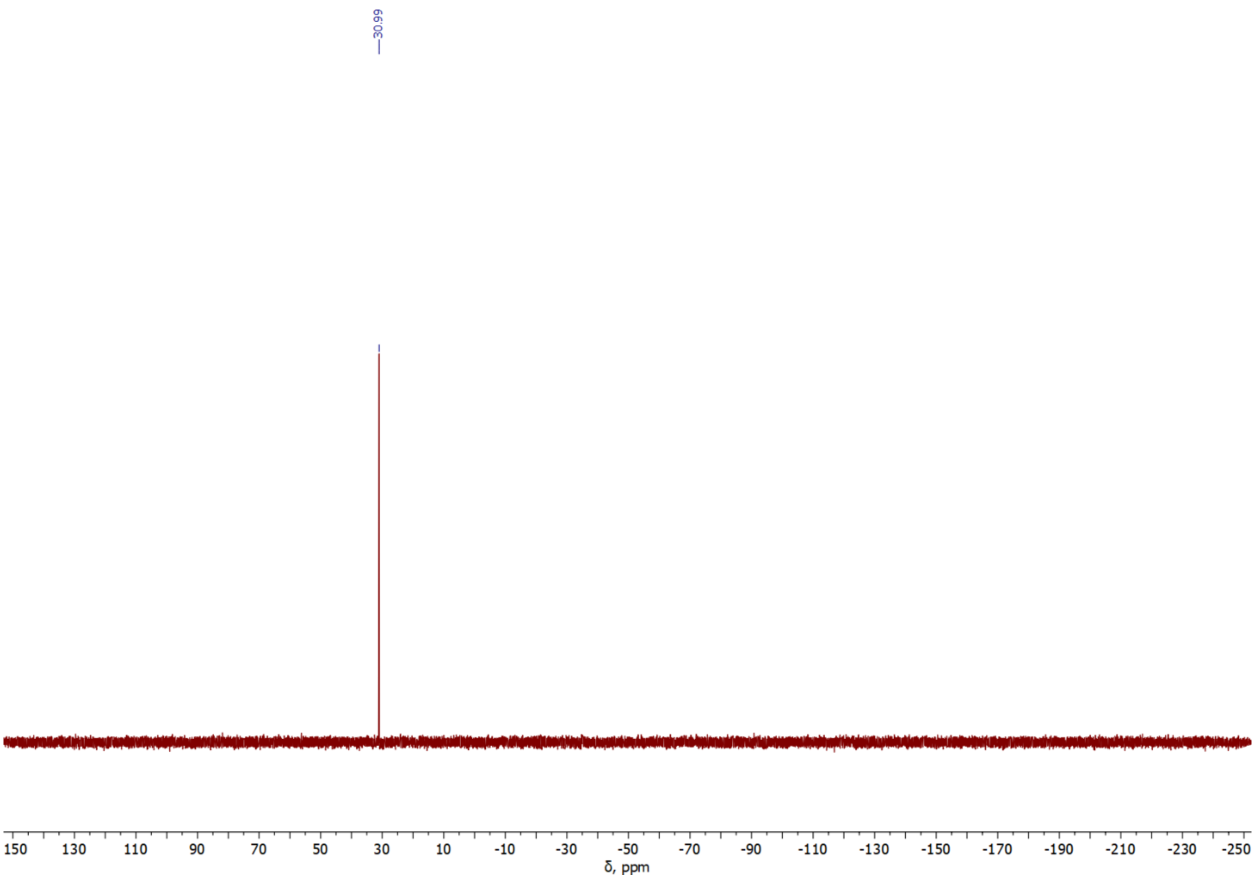

Figure S16. $^{31}P\{^{1}H\}$ NMR (162 MHz, C_6D_6) of **3b-Ir-I**.

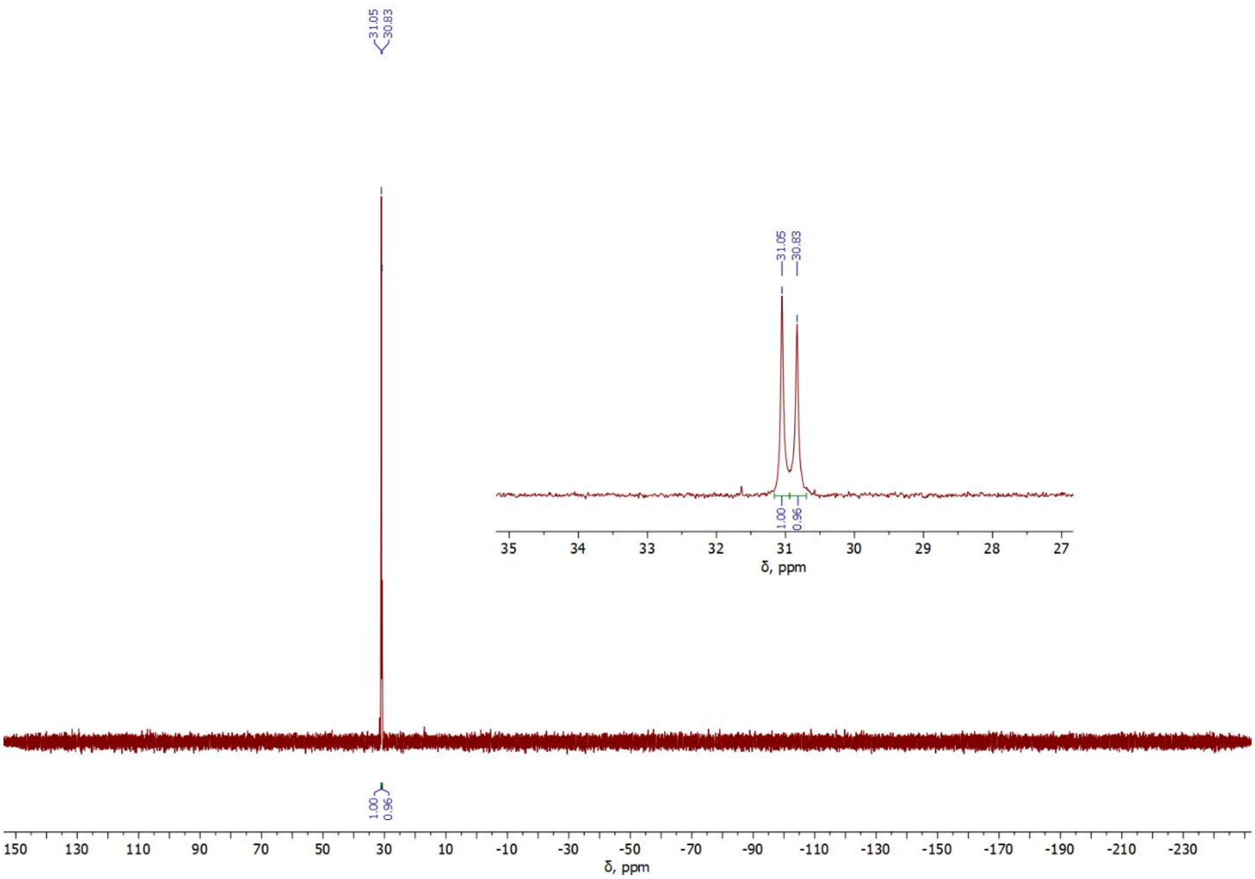

Figure S17. $^{31}P\{^{1}H\}$ NMR (162 MHz, THF-d_8) upon dissolution of **3b-Ir-I** in THF-d_8.

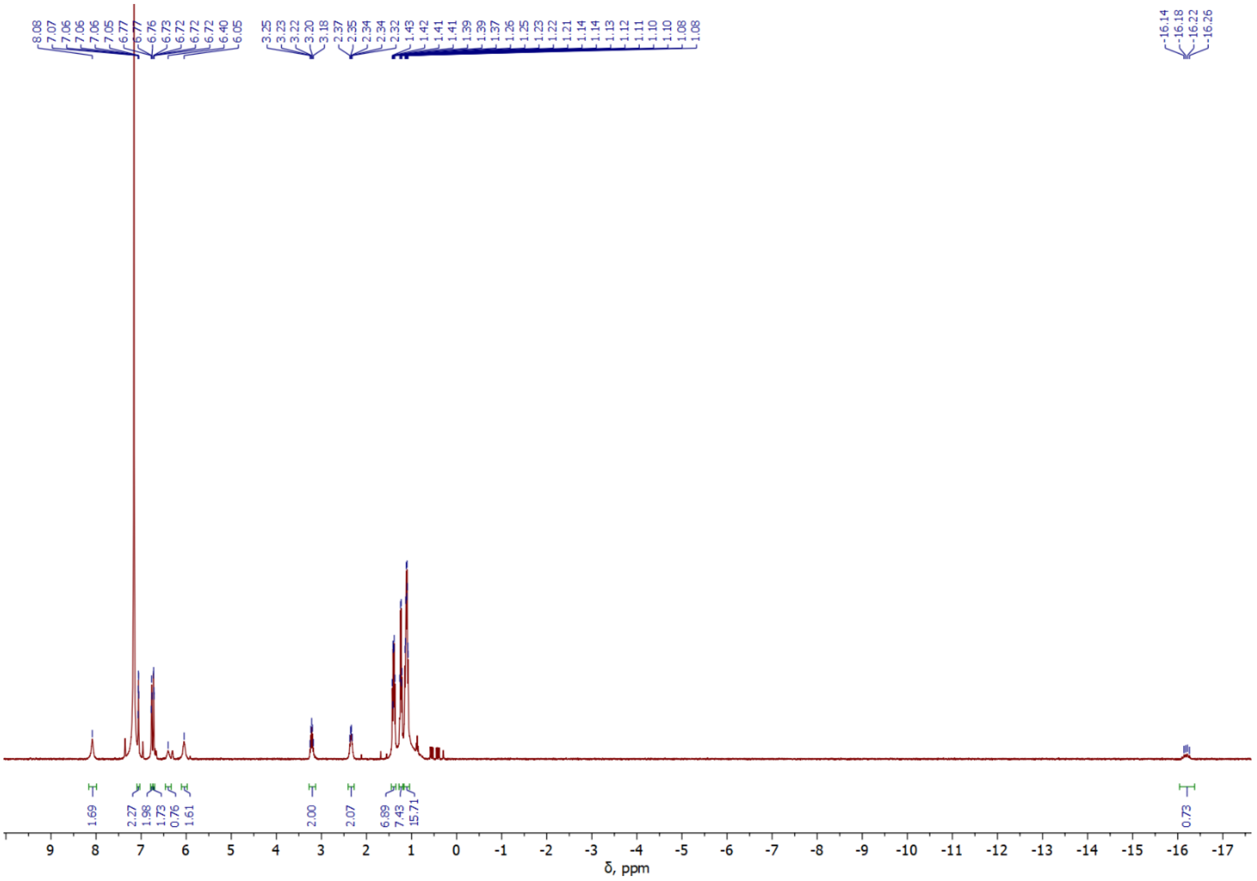

Figure S18. ^{1}H NMR (500 MHz, C_6D_6) of **4b-Rh-I**.

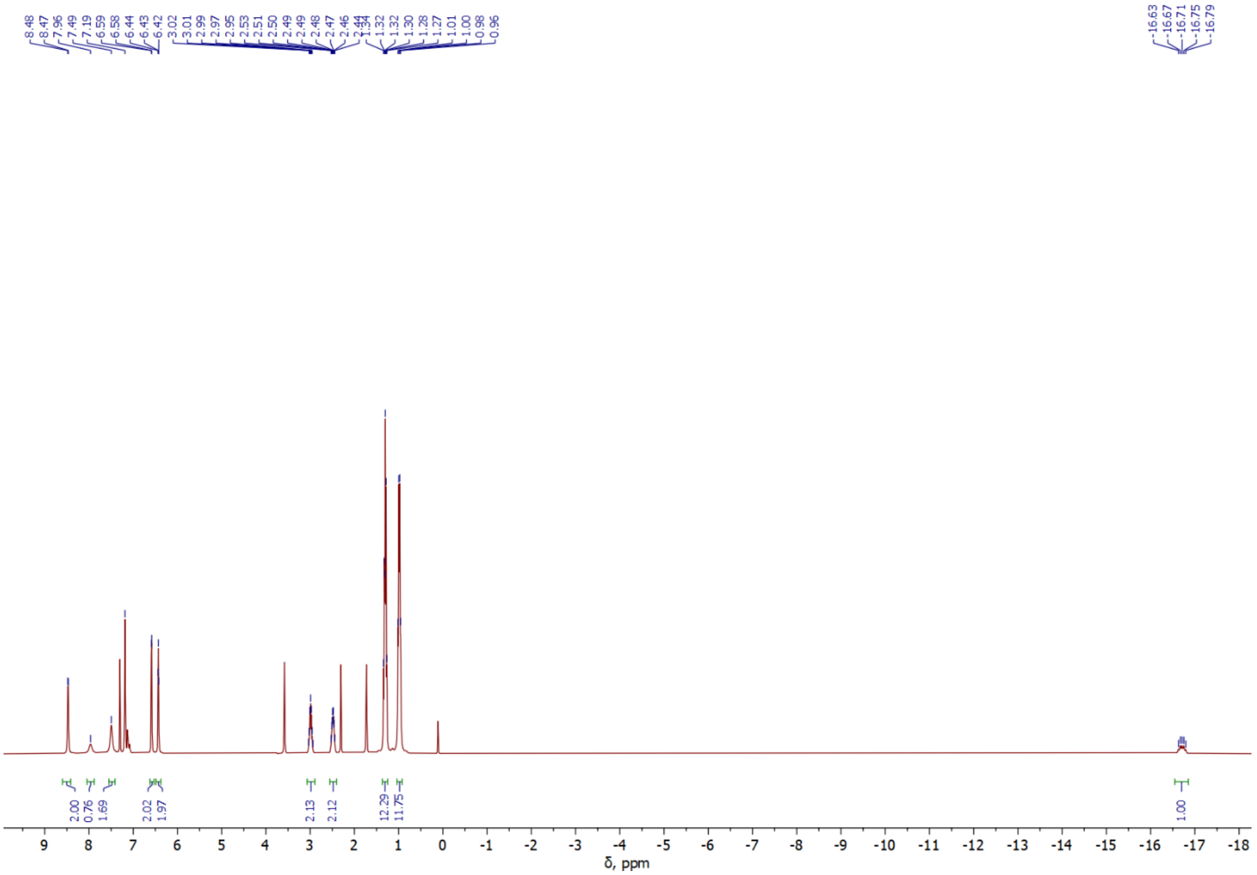

Figure S19. ^{1}H NMR (500 MHz, THF-d_8) of **4b-Rh-I**.

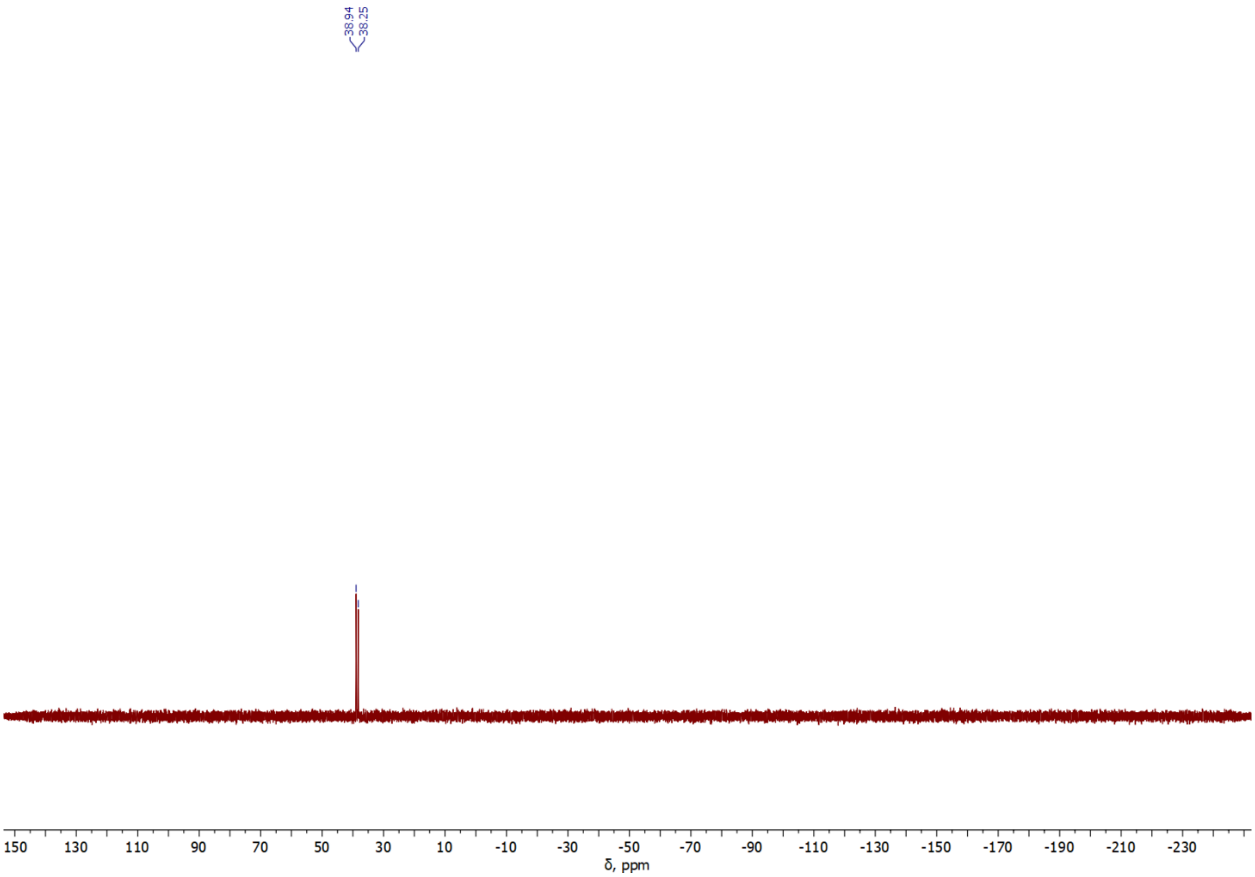

Figure S20. $^{31}P\{^{1}H\}$ NMR (162 MHz, C_6D_6) of **4b-Rh-I**.

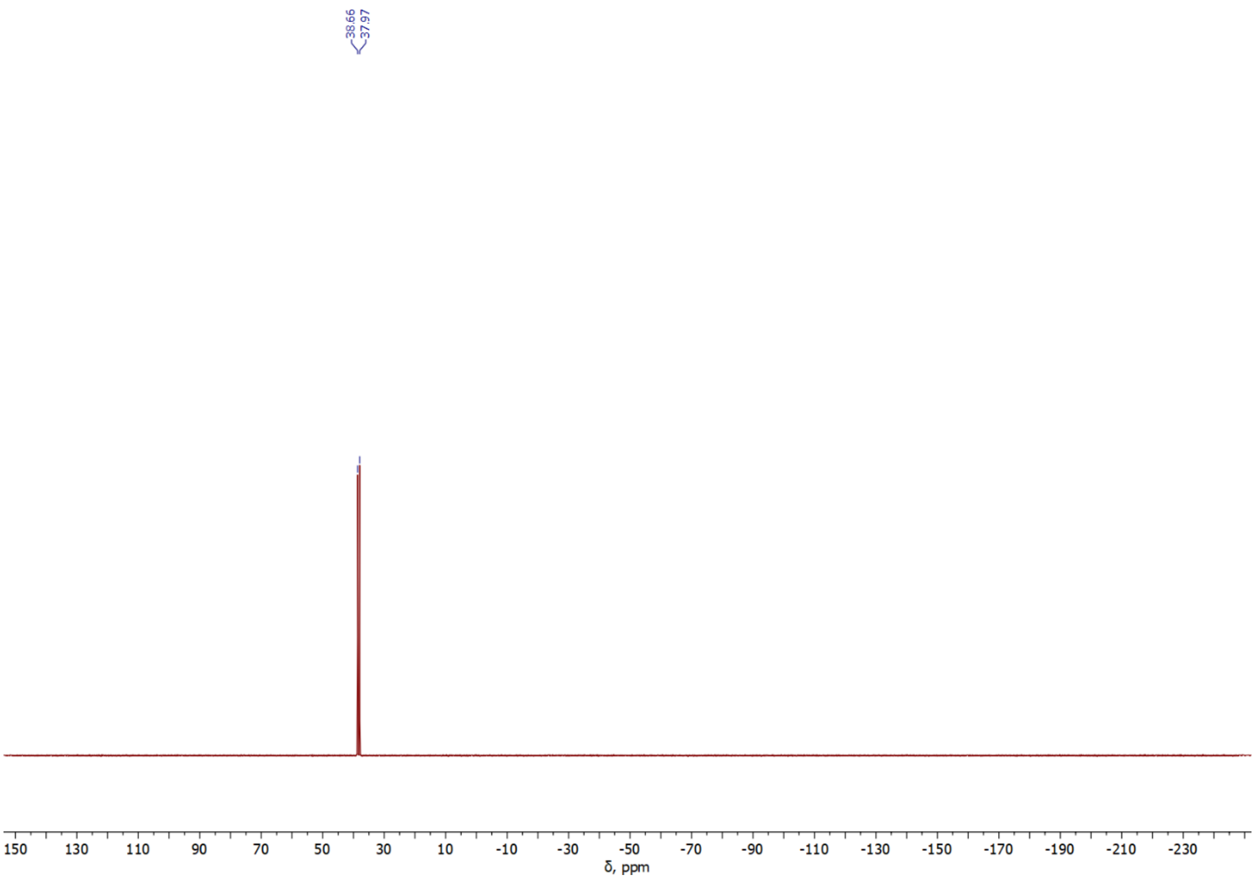

Figure S21. $^{31}P\{^{1}H\}$ NMR (162 MHz, THF-d_8) of **4b-Rh-I**.

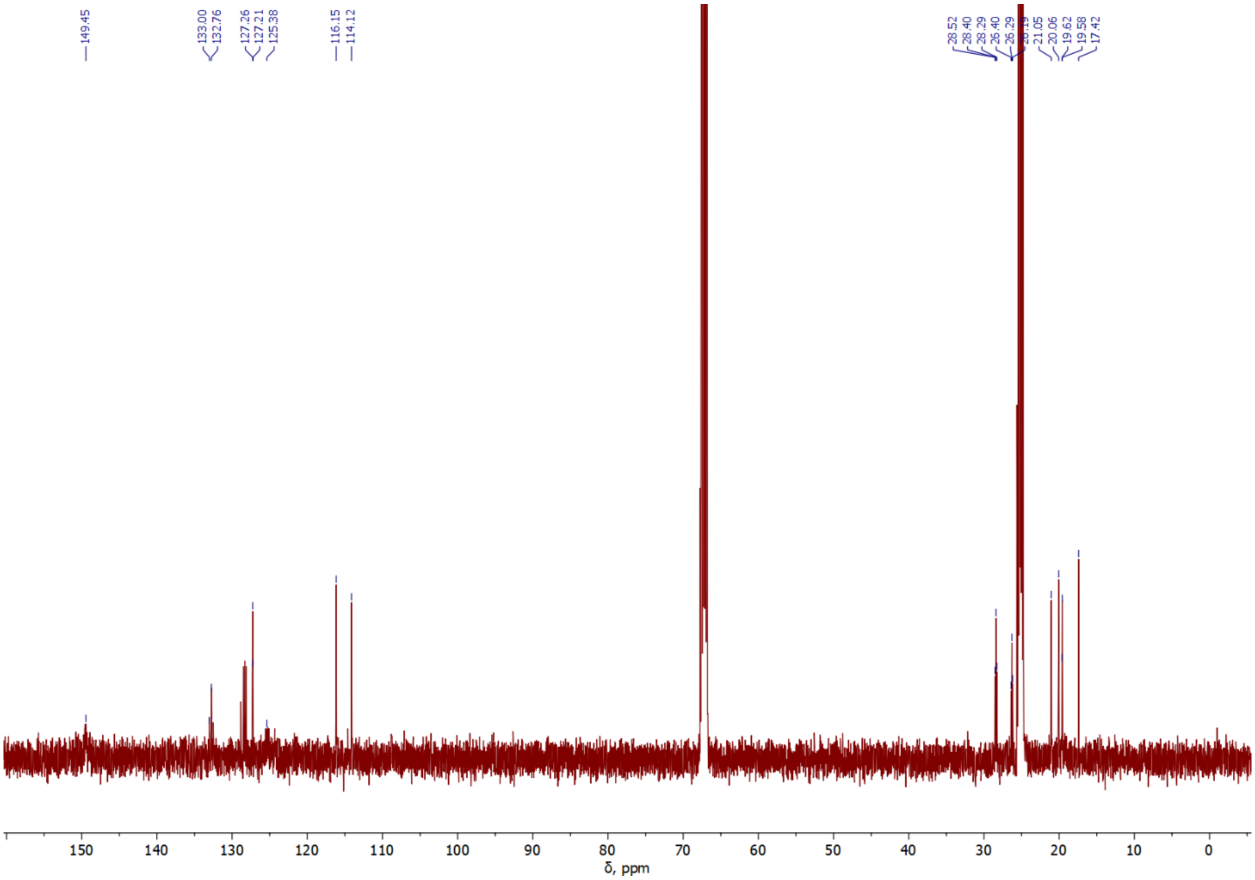

Figure S22. $^{13}C\{^{1}H\}$ NMR (126 MHz, THF-d_8) of **4b-Rh-I**.

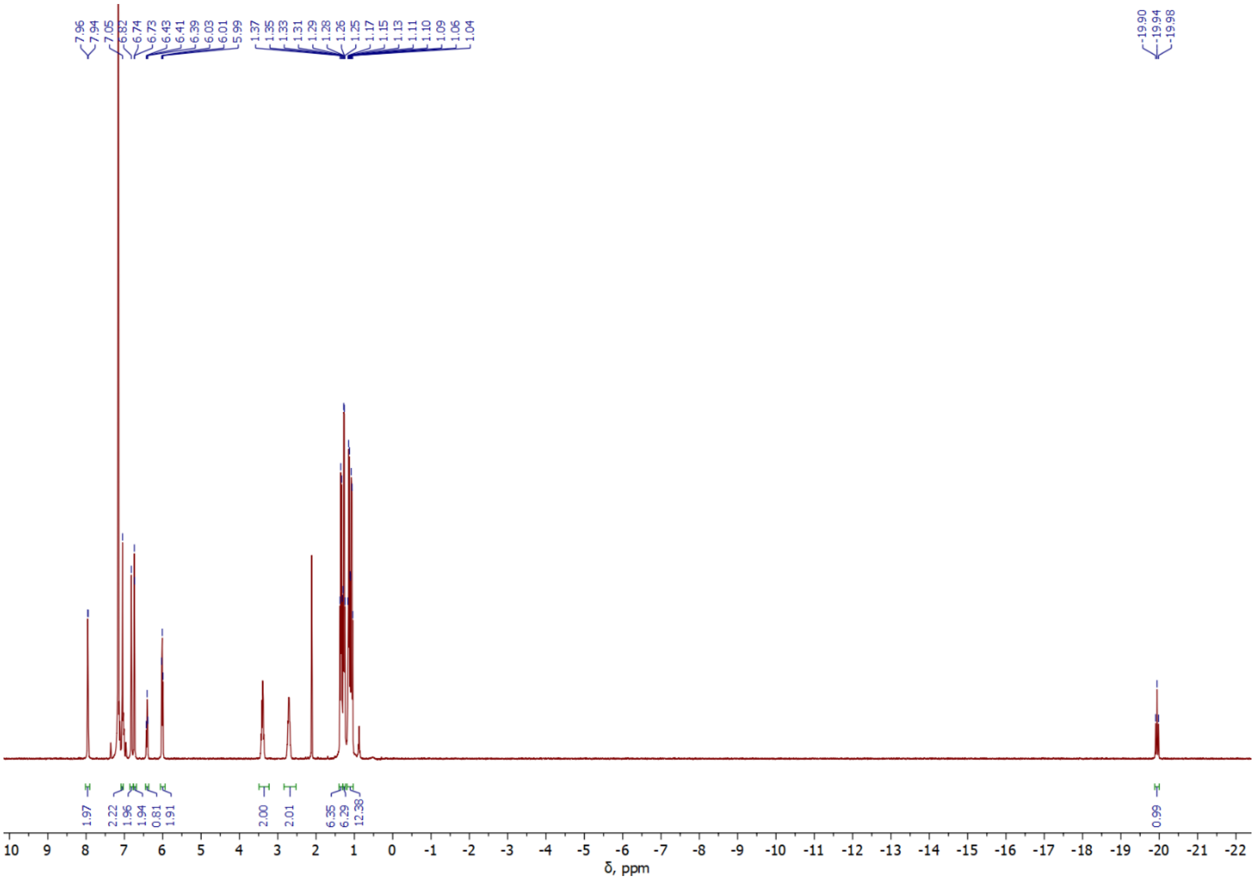

Figure S23. ^{1}H NMR (400 MHz, C_6D_6) of **4b-Ir-I**.

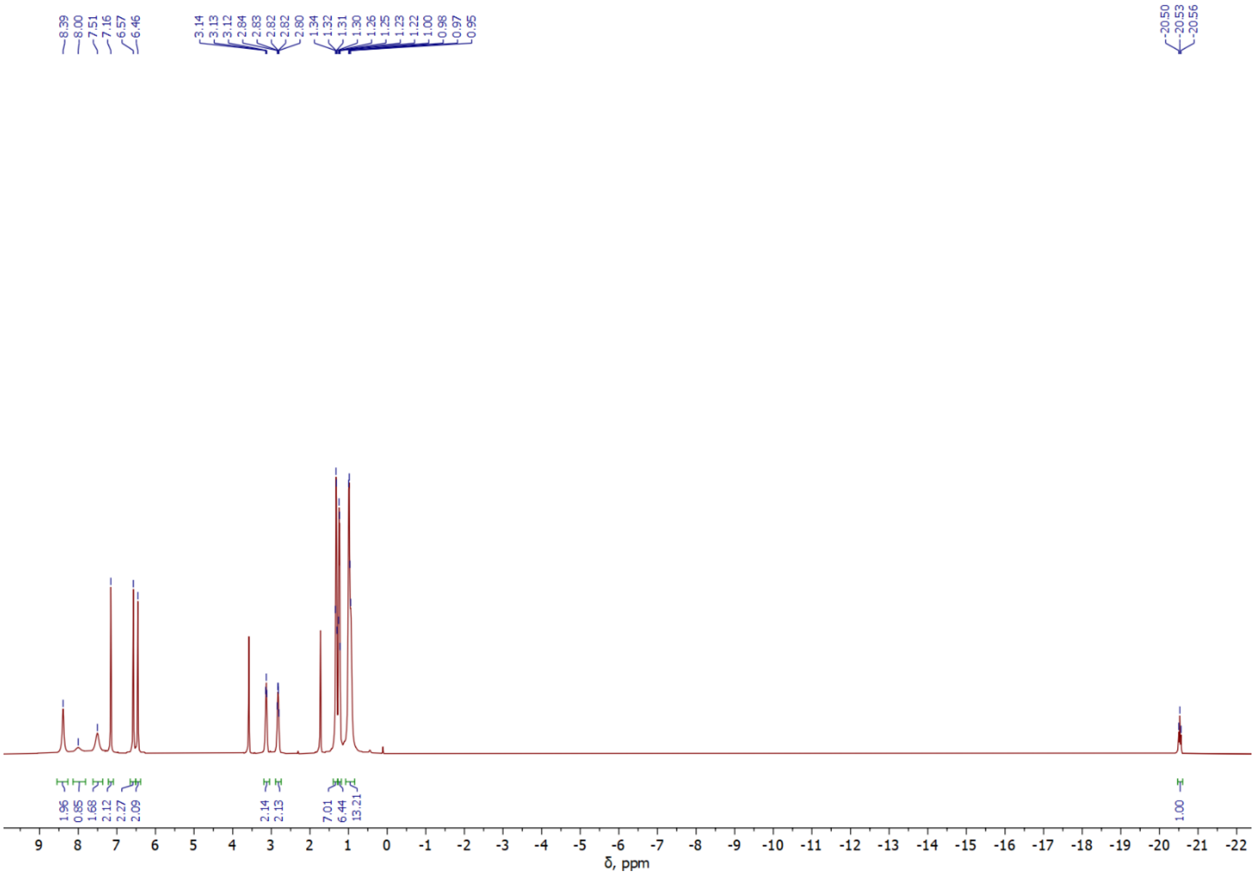

Figure S24. ^{1}H NMR (500 MHz, THF-d_8) of **4b-Ir-I**.

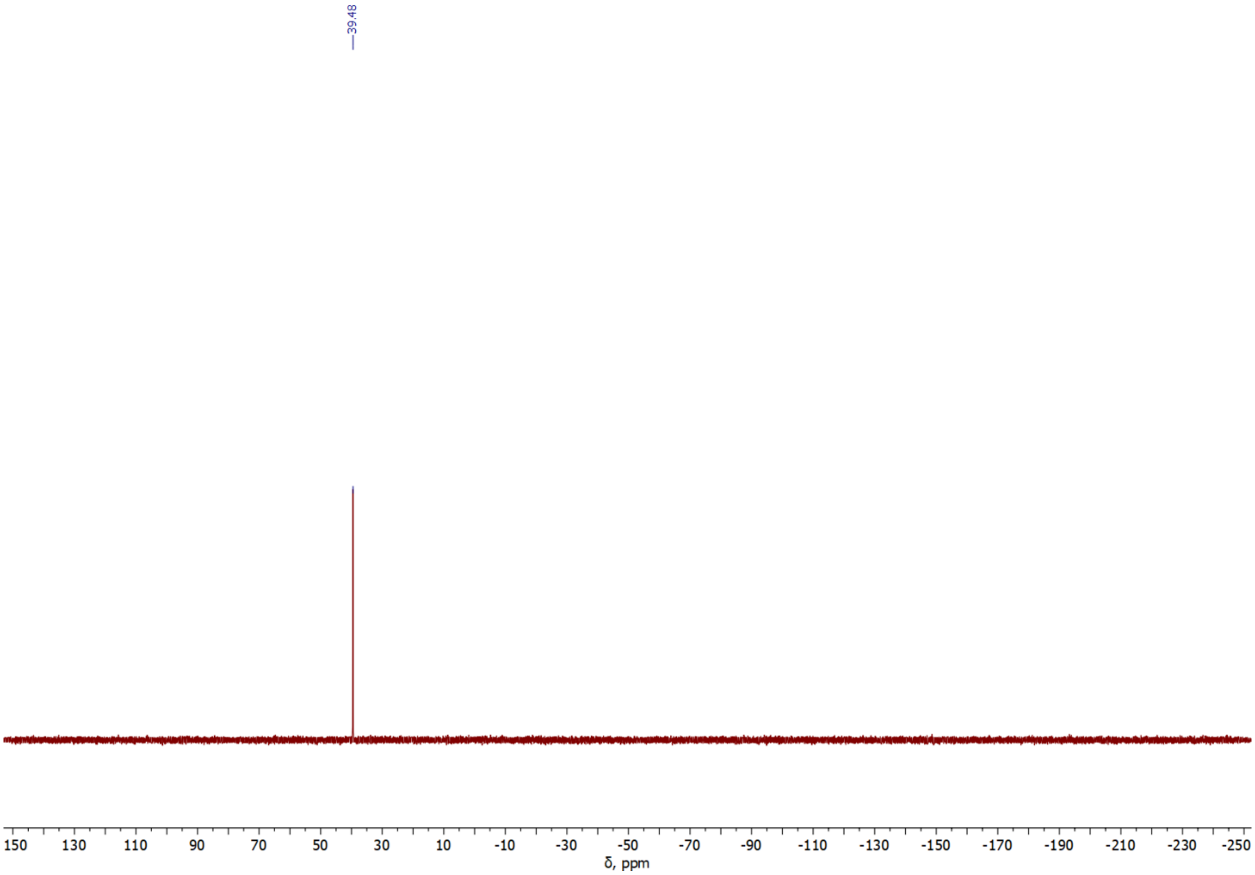

Figure S25. $^{31}P\{^{1}H\}$ NMR (162 MHz, C_6D_6) of **4b-Ir-I**.

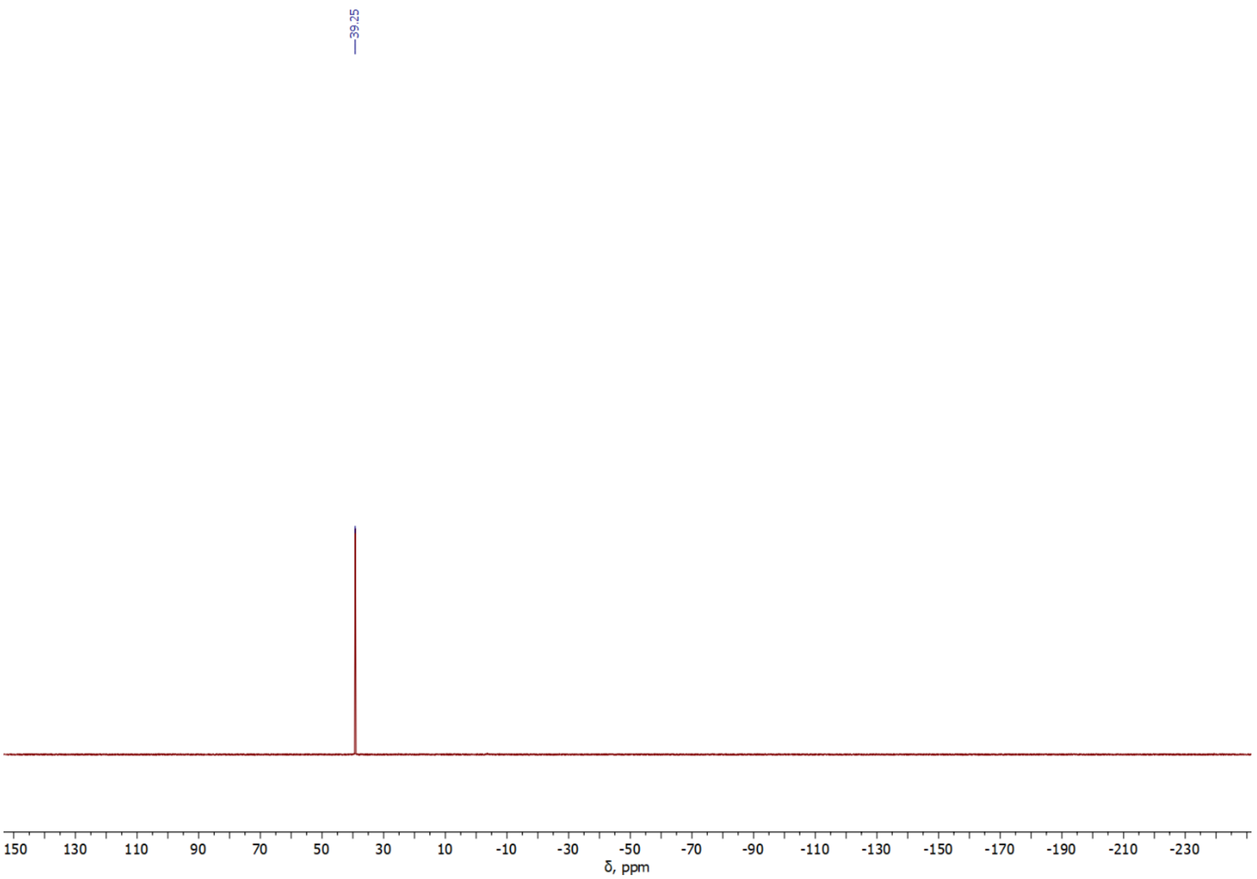

Figure S26. $^{31}P\{^{1}H\}$ NMR (202 MHz, THF-d_8) of **4b-Ir-I**.

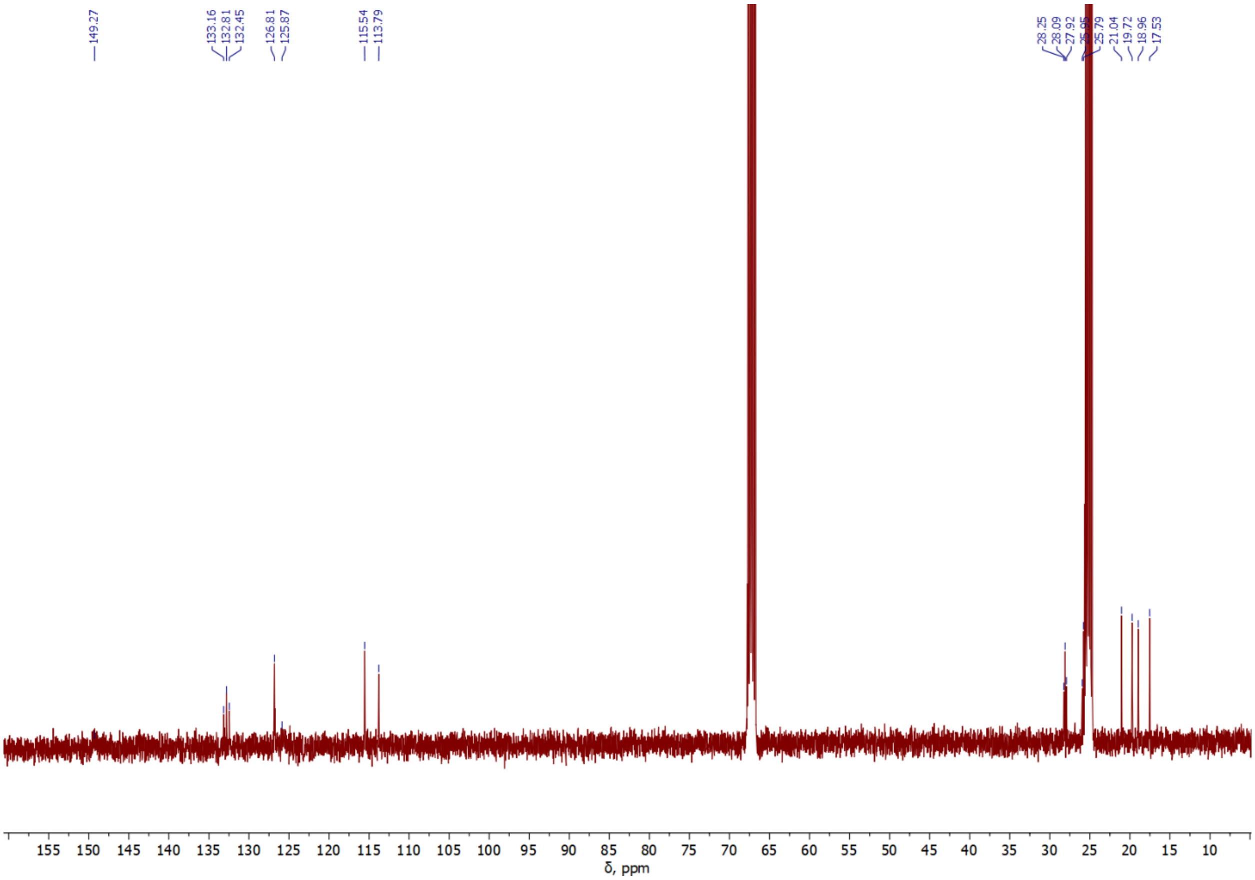

Figure S27. $^{13}C\{^{1}H\}$ NMR (101 MHz, THF-d_8) of **4b-Ir-I**.

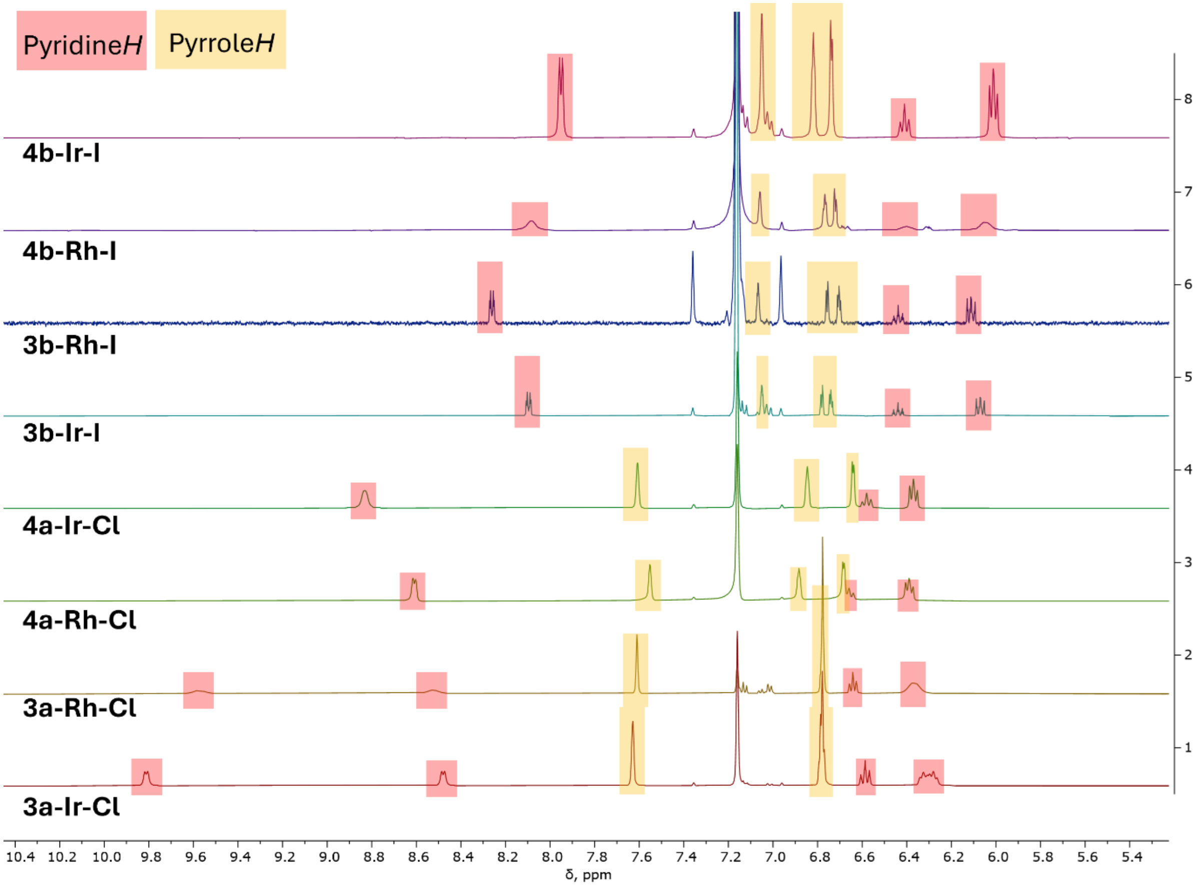

Figure S28. Comparison of ^{1}H NMR spectra for compounds **3-M-X and 4-M-X** in C_6D_6, aromatic region. Solubility of **M-I** compounds in C_6D_6 is poor.

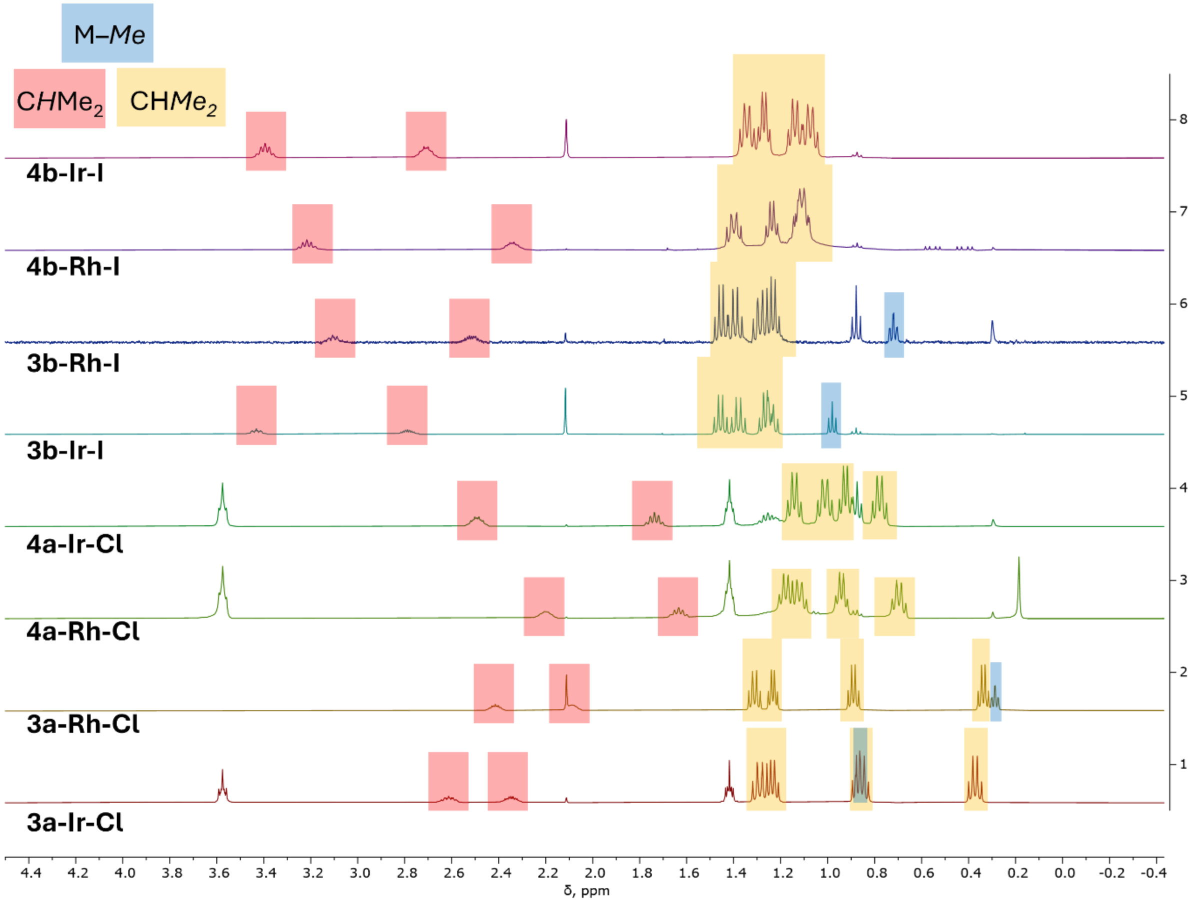

Figure S29. Comparison of ^{1}H NMR for compounds **3-M-X** and **4-M-X** in C_6D_6, aliphatic region. Solubility of **M-I** compounds in C_6D_6 is poor.

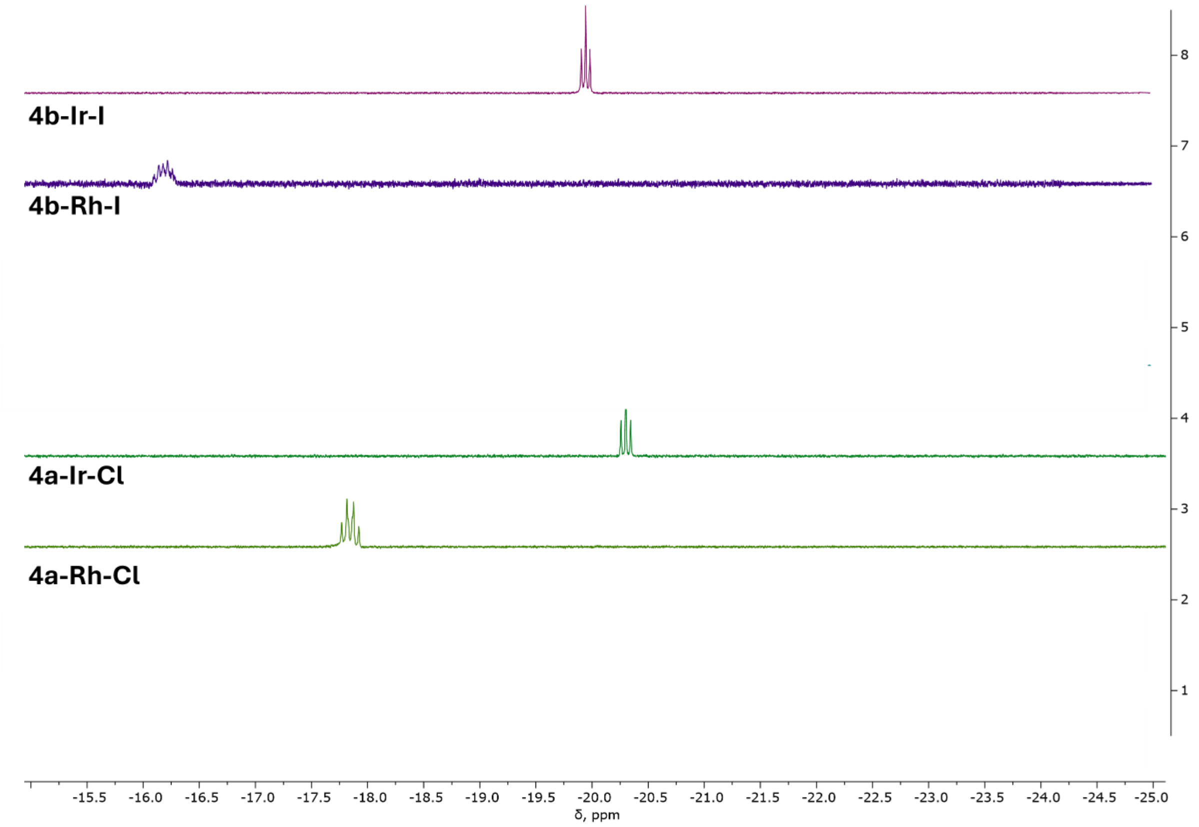

Figure S30. Comparison of ^{1}H NMR for compounds **3-M-X** and **4-M-X** in C_6D_6, hydride region. Solubility of **M-I** compounds in C_6D_6 is poor.

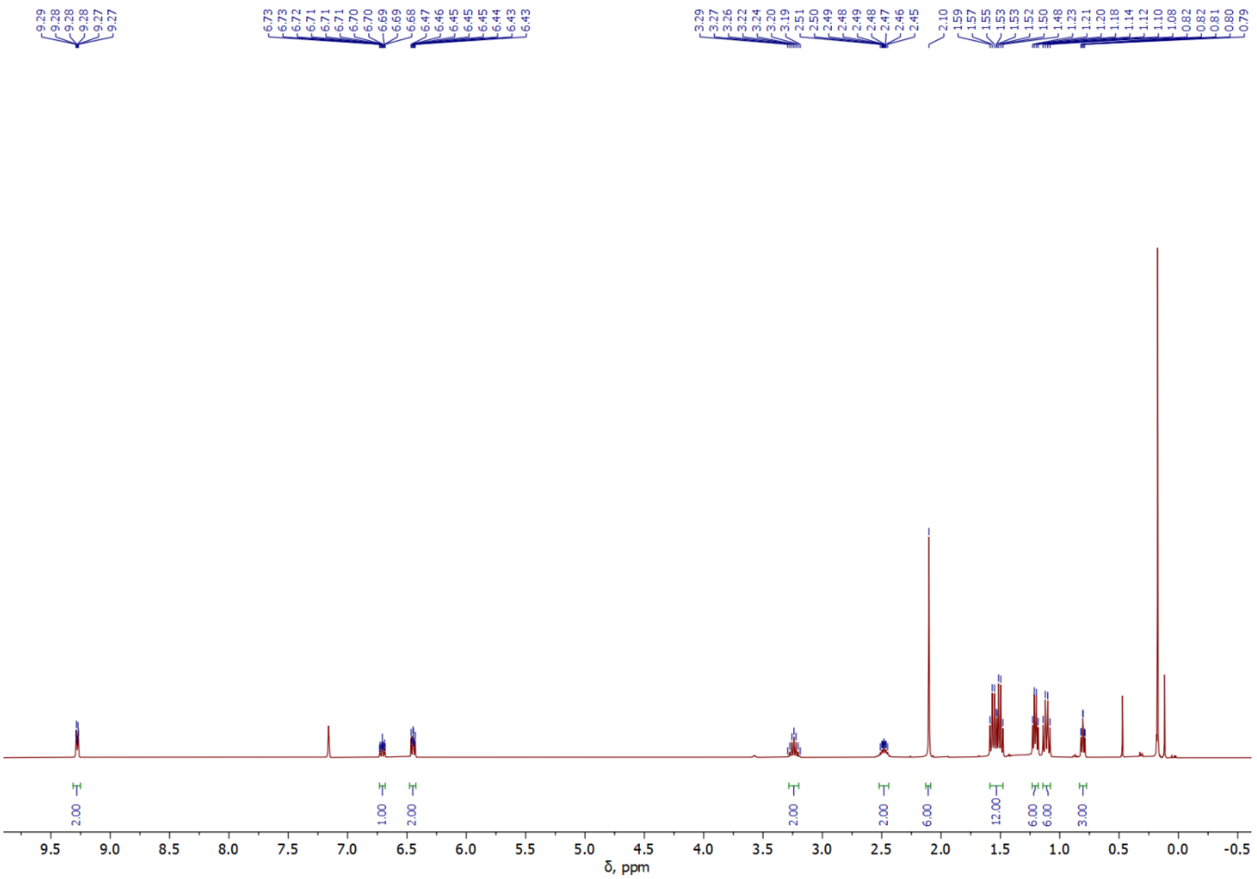

Figure S31. ^{1}H NMR (400 MHz, C_6D_6) spectrum of ^{G}**3b-Rh-I**.

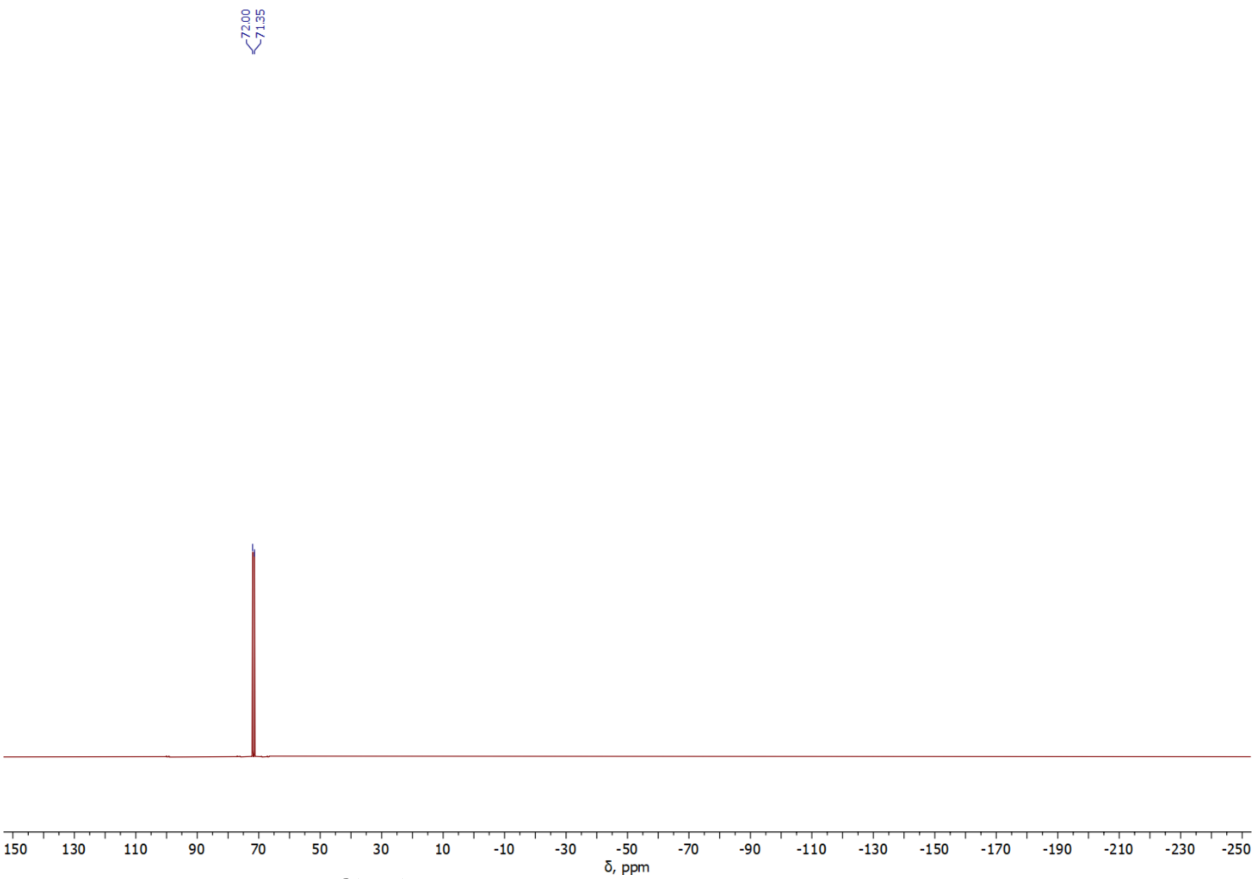

Figure 32. $^{31}P\{^1H\}$ NMR (162 MHz, C_6D_6) spectrum of ^{G}**3b-Rh-I**.

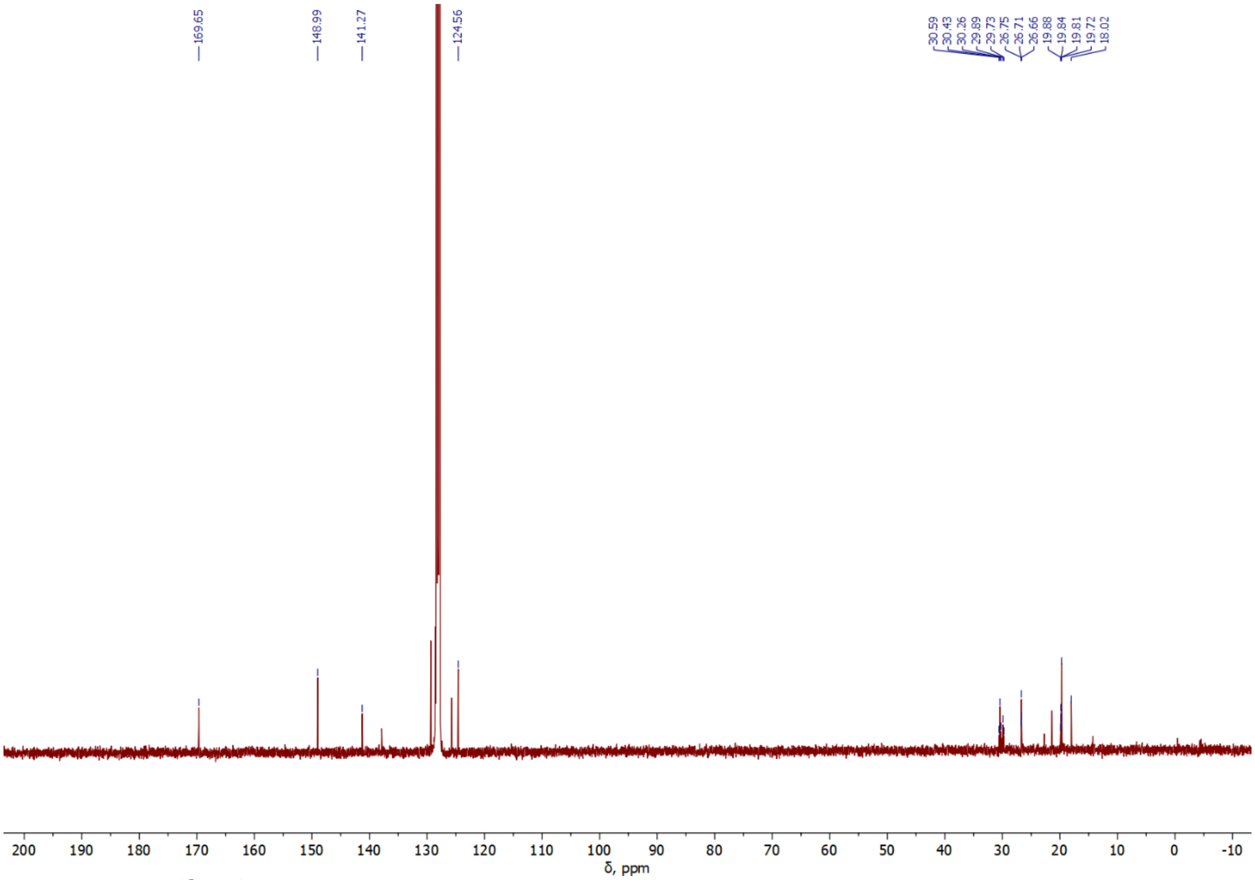

Figure 33. $^{13}C\{^1H\}$ NMR (101 MHz, C_6D_6) spectrum of **^{G}3b-Rh-I**. Sample contains residual toluene.

VI. X-Ray Structural Determination Details

X-ray data collection, solution, and refinement for 3b-Rh-I (CCDC: 2374340).

Data Collection

A Leica M80 microscope was used to identify a suitable single yellow block-shaped crystal of **3b-Rh-I** showing well defined faces with dimensions 0.27 × 0.10 × 0.03 mm^3 from a representative sample of crystals of the same habit. The crystal mounted on a nylon loop was then placed in a cold nitrogen stream (Oxford) maintained at T = 110.0 K.

Crystal screening, unit cell determination, and data collection were carried out using a XtaLAB Synergy, Single source at home/near, Eiger2 1M diffractometer. The diffraction pattern was indexed and the total number of runs and images was based on the strategy calculation from the program CrysAlisPro system.[16] Data were measured using w scans with Ag K_a radiation. Data was collected to a maximum resolution of Q = 27.864° (0.60 Å). The unit cell was refined using CrysAlisPro 1.171.43.98a[16] on 43436 reflections, 70 % of the observed reflections.

Data Reduction, Structure Solution, and Refinement

Integrated Intensity information for each reflection was obtained by reduction of data frames using CrysAlisPro 1.171.43.98a.[16] The final completeness is 99.70 % out to 27.864° in Q. A gaussian absorption correction was performed using CrysAlisPro 1.171.43.98a.[16] Numerical absorption correction based on gaussian integration over a multifaceted crystal model Empirical absorption correction using spherical harmonics, implemented in SCALE3 ABSPACK scaling algorithm. The absorption coefficient m of this material is 0.942 mm^{-1} at this wavelength (l = 0.56087Å) and the minimum and maximum transmissions are 0.766 and 1.000.

Systematic reflection conditions and statistical tests of the data suggested the space group *P*-1 (# 2) and was confirmed by ShelXT[17] structure solution program using dual methods. The structure was refined by full matrix least squares minimisation on F^2 using version 2019/1 of ShelXL 2019/1.[18] All non-hydrogen atoms were refined anisotropically. Hydrogen atom positions were calculated geometrically and refined using the riding model.

VII. SI References

(1) Herde, J. L.; Lambert, J. C.; Senoff, C. V.; Cushing, M. A. Cyclooctene and 1,5-Cyclooctadiene Complexes of Iridium(I). *Inorg. Synth.* **1974**, *15*, 18−20

(2) Giordano, G.; Crabtree, R. H.; Heintz, R. M.; Forster, D.; Morris, D. E. Di-μ-Chloro-Bis(H_4-1,5-Cyclooctadiene)-Dirhodium(I). *Inorg. Synth.* **1990**, *28*, 88−90.

(3) Lai, Q.; Cosio, M. N.; Ozerov, O. V. Ni Complexes of an Alane/Tris(Phosphine) Ligand Built around a Strongly Lewis Acidic Tris(N-Pyrrolyl)Aluminum. *Chem. Commun* **2020**, *56*, 14845–14848.

(4) Frisch, M. J.; Trucks, G. W.; Schlegel, H. B.; Scuseria, G. E.; Robb, M. A.; Cheeseman, J. R.; Scalmani, G.; Barone, V.; Petersson, G. A.; Nakatsuji, H.; Li, X.; Caricato, M.; Marenich, A. V.; Bloino, J.; Janesko, B. G.; Gomperts, R.; Mennucci, B.; Hratchian, H. P.; Ortiz, J. V.; Izmaylov, A. F.; Sonnenberg, J. L.; Williams-Young, D.; Ding, F.; Lipparini, F.; Egidi, F.; Goings, J.; Peng, B.; Petrone, A.; Henderson, T.; Ranasinghe, D.; Zakrzewski, V. G.; Gao, J.; Rega, N.; Zheng, G.; Liang, W.; Hada, M.; Ehara, M.; Toyota, K.; Fukuda, R.; Hasegawa, J.; Ishida, M.; Nakajima, T.; Honda, Y.; Kitao, O.; Nakai, H.; Vreven, T.; Throssell, K.; Montgomery, J. A., Jr.; Peralta, J. E.; Ogliaro, F.; Bearpark, M. J.; Heyd, J. J.; Brothers, E. N.; Kudin, K. N.; Staroverov, V. N.; Keith, T. A.; Kobayashi, R.; Normand, J.; Raghavachari, K.; Rendell, A. P.; Burant, J. C.; Iyengar, S. S.; Tomasi, J.; Cossi, M.; Millam, J. M.; Klene, M.; Adamo, C.; Cammi, R.; Ochterski, J. W.; Martin, R. L.; Morokuma, K.; Farkas, O.; Foresman, J. B.; Fox, D. J. Gaussian, Inc., Wallingford CT, 2016. Gaussian 16, Revision C.01, 2016.

(5) Zhao, Y.; Truhlar, D. G. The M06 Suite of Density Functionals for Main Group Thermochemistry, Thermochemical Kinetics, Noncovalent Interactions, Excited States, and Transition Elements: Two New Functionals and Systematic Testing of Four M06-Class Functionals and 12 Other Functionals. *Theor. Chem. Acc.* **2008**, *120*, 215–241.

(6) Andrae, D.; Häuβermann, U.; Dolg, M.; Stoll, H.; Preuβ, H. Energy-Adjustedab Initio Pseudopotentials for the Second and Third Row Transition Elements. *Theor. Chim. Acta* **1990**, *77*, 123–141.

(7) Francl, M. M.; Pietro, W. J.; Hehre, W. J.; Binkley, J. S.; Gordon, M. S.; DeFrees, D. J.; Pople, J. A. Self-consistent Molecular Orbital Methods. XXIII. A Polarization-type Basis Set for Second-row Elements. *J. Chem. Phys.* **1982**, *77*, 3654–3665.

(8) Krishnan, R.; Binkley, J. S.; Seeger, R.; Pople, J. A. Self-consistent Molecular Orbital Methods. XX. A Basis Set for Correlated Wave Functions. *J. Chem. Phys.* **1980**, *72*, 650–654.

(9) McLean, A. D.; Chandler, G. S. Contracted Gaussian Basis Sets for Molecular Calculations. I. Second Row Atoms, Z=11–18. *J. Chem. Phys.* **1980**, *72*, 5639–5648.

(10) Marenich, A. V.; Cramer, C. J.; Truhlar, D. G. Universal Solvation Model Based on Solute Electron Density and on a Continuum Model of the Solvent Defined by the Bulk Dielectric Constant and Atomic Surface Tensions. *J. Phys. Chem. B* **2009**, *113*, 6378–6396.

(11) Clark, T.; Chandrasekhar, J.; Spitznagel, G. W.; Schleyer, P. V. R. Efficient Diffuse Function-Augmented Basis Sets for Anion Calculations. III. The 3-21+G Basis Set for First-Row Elements, Li–F. *J. Comput. Chem.* **1983**, *4*, 294–301.

(12) Spitznagel, G. W.; Clark, T.; von Ragué Schleyer, P.; Hehre, W. J. An Evaluation of the Performance of Diffuse Function-Augmented Basis Sets for Second Row Elements, Na-Cl. *J. Comput. Chem.* **1987**, *8*, 1109–1116.

(13) Glendening, E. D.; Landis, C. R.; Weinhold, F. NBO 6.0: Natural Bond Orbital Analysis Program. *J. Comput. Chem.* **2013**, *34*, 1429–1437.

(14) Legault, C. Y. CYLview20, 2020.

(15) Nguyen, V. T.; Lai, Q.; Witayapaisitsan, N.; Bhuvanesh, N.; Surawatanawong, P.; Ozerov, O. V. Migration of Hydride, Methyl, and Chloride Ligands between Al and M in (PAlP)M Pincer Complexes (M = Rh or Ir). *Organometallics* **2023**, *42*, 3120–3129.

(16) Rigaku Oxford Diffraction. *CrysAlisPro Software System*.

(17) Sheldrick, G. M. SHELXT – Integrated Space-Group and Crystal-Structure Determination. *Acta Cryst A* **2015**, *71*, 3–8.

(18) Sheldrick, G. M. Crystal Structure Refinement with SHELXL. *Acta Cryst C* **2015**, *71*, 3–8.
